# Heart Failure with Reduced and Mildly Reduced Ejection Fraction: A Network Interpretive Framework of Mechanisms, Phenotypes, and Therapeutic Response

**DOI:** 10.3390/ijms27146370

**Published:** 2026-07-17

**Authors:** Beata Krasińska, Giuseppe Maria Raffa, Calogera Pisano, Vincenzo Nuzzi, Paolo Manca, Krzysztof J. Filipiak, Mansur Rahnama, Mariusz Kowalewski, Zbigniew Krasiński, Piotr Suwalski, Sebastian Mertowski, Paulina Mertowska, Ewelina Grywalska, Tomasz Urbanowicz

**Affiliations:** 1 Department of Hypertensiology, Angiology, and Internal Medicine, Poznan University of Medical Sciences, 61-848 Poznan, Poland; 2 Department of Research, IRCCS-ISMETT (Mediterranean Institute for Transplantation and Specialized Therapies), 90127 Palermo, Italy; 3 Department of Precision Medicine in Medical Surgical and Critical Area (Me.Pre.C.C.), University of Palermo, 90134 Palermo, Italy; 4 Department of Clinical Cardiology and Heart Failure, IRCCS ISMETT (Mediterranean Institute for Transplantation and Advanced Specialized Therapies), 90127 Palermo, Italy; 5 The Centre of Postgraduate Medical Education, 99/103 Marymoncka Street, 01-813 Warsaw, Poland; 6 Department of Dental Surgery, Medical University of Lublin, 6 Chodźki Street, 20-093 Lublin, Poland; 7 Department of Cardiac Surgery, Center of Postgraduate Medical Education, Central Clinical Hospital of the Ministry of Interior, 02-507 Warszawa, Poland; 8 Department of Vascular, Endovascular Surgery, Angiology and Phlebology, Poznan University of Medical Science, ½ Dluga Street, 61-848 Poznan, Poland; 9 Department of Experimental Immunology, Medical University of Lublin, 20-093 Lublin, Poland; 10 Cardiac Surgery and Transplantology Department, Poznan University of Medical Sciences, ½ Długa, 61-848 Poznan, Poland

**Keywords:** heart failure, HFrEF, HFmrEF, ejection fraction, network biology, mechanism-guided therapy

## Abstract

Heart failure (HF) classification is still primarily based on left ventricular ejection fraction, even though this parameter only partially reflects the biological mechanisms determining disease progression and therapeutic response. The aim of this review was to present a conceptual interpretive framework that analyzes the HFrEF (heart failure with reduced ejection fraction) and HFmrEF (heart failure with mildly reduced ejection fraction) phenotypes through the lens of the network organization of pathophysiological processes. This review integrates data on molecular and cellular mechanisms, clinical phenotypes, clinical trial results, and therapeutic recommendations. Particular attention was paid to the concepts of network coherence, pathway dominance, and the relationship between the disease’s biological architecture and treatment response. The proposed conceptual framework suggests that HFrEF is more often characterized by a relatively coherent pathophysiological architecture, in which neurohormonal activation, disturbances in calcium metabolism, mitochondrial dysfunction, and extracellular matrix remodeling constitute mutually reinforcing processes. HFmrEF, on the other hand, is presented as a heterogeneous category, encompassing patients with partial improvement of previous systolic dysfunction, patients progressing towards HFrEF, and HFpEF (heart failure with preserved ejection fraction)—like phenotypes associated with inflammation, endothelial dysfunction, microcirculatory disturbances, and metabolic dysregulation. In this approach, therapeutic response depends on whether the targeted pathway occupies a central position in the disease network. This review proposes a hypothesis-generating conceptual framework that complements, rather than replaces, the current ejection fraction-based classification of heart failure. Although the proposed framework requires prospective validation, it may facilitate a more mechanistic interpretation of HF phenotypes, support future biologically informed therapeutic strategies, and stimulate the design of mechanistically oriented clinical studies.

## 1. Introduction

Heart failure (HF) remains a leading cause of morbidity, hospitalization, and mortality worldwide, and its classification is still largely based on left ventricular ejection fraction (LVEF) [1,2,3,4]. Although LVEF is a readily available, clinically practical parameter for patient selection for treatment, it does not fully capture the biological complexity of HF. It is an endpoint measure of ventricular function that results from the interaction among myocardial contractility, left ventricular geometry, hemodynamic load, and systemic mechanisms, rather than a direct marker of a specific pathophysiological process [5,6,7,8,9].

The traditional classification of HF into phenotypes based on LVEF, including HF with reduced ejection fraction (HFrEF), HF with mildly reduced ejection fraction (HFmrEF), and HF with preserved ejection fraction (HFpEF), has played a key role in clinical trial design and treatment recommendations [9,10,11]. The clinical status, echocardiographic results, and traditional serum biomarker concentrations constitute the core of the patients’ profiles. Circulating biomarkers [12,13] provide valuable information on myocardial injury, wall stress, inflammation, fibrosis, and neurohormonal activation; they should not be regarded as direct measures of the underlying biological network. Most currently available biomarkers reflect the integrated consequences of multiple interacting pathophysiological processes and are influenced by numerous cardiac and extracardiac conditions. Therefore, throughout this review, biomarkers are considered complementary indicators that may contribute to biological phenotyping when interpreted together with imaging findings, clinical characteristics, and disease trajectory, rather than as independent measures of pathway dominance.

According to the contemporary European Society of Cardiology and American Heart Association/Heart Failure Society of America guidelines, heart failure is classified according to left ventricular ejection fraction into heart failure with reduced ejection fraction (HFrEF; LVEF ≤ 40%), heart failure with mildly reduced ejection fraction (HFmrEF; LVEF 41–49%), and heart failure with preserved ejection fraction (HFpEF; LVEF ≥ 50%) [14,15,16]. In addition, heart failure with improved ejection fraction (HFimpEF) refers to patients with a prior LVEF ≤ 40% who subsequently improve by at least 10 percentage points to an LVEF > 40% following treatment or recovery of myocardial function. These categories remain the foundation of current guideline-directed diagnosis and management, although growing evidence suggests they do not fully capture the underlying biological heterogeneity of heart failure.

However, it is increasingly recognized that similar LVEF values may result from distinct molecular, cellular, and structural mechanisms, and similar biological processes may occur across LVEF categories. This limits the usefulness of classification based solely on EF in predicting disease course and treatment response [17,18,19,20].

HFrEF is a phenotype in which systolic dysfunction is often associated with a more coherent pathophysiological architecture. In many patients, chronic neurohormonal activation, involving the renin–angiotensin–aldosterone system and the sympathetic nervous system, plays a dominant role, driving left ventricular remodeling, fibrosis, calcium dysfunction, mitochondrial dysfunction, and oxidative stress. This relatively mechanistic coherence may partially explain the reproducible clinical benefits of treatments targeting upstream regulatory pathways observed in numerous clinical trials in HFrEF [21,22,23,24,25].

HFmrEF presents a different challenge, as it does not appear to be a homogeneous biological entity but rather a category encompassing distinct disease trajectories. This group may include patients with partial recovery from previous HFrEF, patients in the early phase of progression towards systolic dysfunction, and patients with a phenotype similar to HFpEF, in whom inflammation, endothelial dysfunction, microcirculation abnormalities, metabolic disorders, and comorbidities are more prominent. This heterogeneity complicates the interpretation of clinical trial results and may explain the more variable and phenotype-dependent therapeutic response observed in HFmrEF [26,27].

In this context, it may be useful to view HF as a network disorder, in which the course of the disease depends not on a single pathway but on the organization of interconnected biological processes [28]. In this paper, the concept of network coherence is understood as the degree to which disease behavior is governed by a limited number of dominant, hierarchically organized pathways whose modulation leads to predictable biological and clinical outcomes. However, it should be emphasized that this is a conceptual, hypothesis-generating construct, not a currently validated biomarker or a formal quantitative indicator.

This approach allows us to interpret the differences between HFrEF and HFmrEF not only through the lens of LVEF values but also by considering the degree of dominance of individual pathways, the organization of the pathophysiological network, and the presence of intermediate phenotypes. It may also help us understand why therapies targeting classic neurohormonal pathways demonstrate stronger and more reproducible effects in HFrEF, whereas in HFmrEF, their effectiveness depends more on the underlying biology of the phenotype. Despite its practical value, LVEF should be regarded primarily as a phenotypic descriptor rather than a direct indicator of the underlying biological mechanisms.

The purpose of this review is to present a conceptual interpretive framework that analyzes HF with reduced and mildly reduced ejection fraction from the perspective of the network organization of pathophysiological mechanisms.

### 1.1. Why EF-Based Classification Is Reaching Its Biological Limits

The classification of heart failure according to left ventricular ejection fraction has profoundly influenced clinical research, therapeutic development, and guideline formulation over the last three decades. The distinction between HFrEF, HFmrEF, and HFpEF has enabled the creation of relatively homogeneous study populations and has facilitated the translation of trial results into clinical practice. Nevertheless, increasing mechanistic insight suggests that EF (ejection fraction)-based classification represents a functional description rather than a biological taxonomy of disease.

Ejection fraction is an integrated physiological parameter influenced by myocardial contractility, ventricular geometry, loading conditions, valvular function, vascular stiffness, heart rhythm, and systemic neurohormonal activity. Consequently, similar EF values may emerge from fundamentally different pathophysiological processes. Conversely, patients belonging to different EF categories may share substantial overlap in molecular pathways, remodeling patterns, and biomarker profiles.

This limitation has become increasingly apparent in contemporary clinical trials. Therapeutic responses frequently vary within the same EF category, whereas some interventions demonstrate benefits across a broad spectrum of ventricular function. Such observations challenge the traditional assumption that EF alone adequately captures the biological substrate of disease.

The emerging concept of heart failure as a systems-level disorder provides an alternative perspective. Rather than viewing HF as a consequence of a single dominant abnormality, this framework considers disease progression as the result of dynamic interactions among neurohormonal activation, inflammation, metabolic stress, mitochondrial dysfunction, endothelial injury, extracellular matrix remodeling, and organ-to-organ communication. In this context, EF may be interpreted as a downstream phenotypic consequence of network behavior rather than a direct indicator of network architecture itself.

Despite these limitations, the present review intentionally retains the conventional EF-based classification as its organizational framework. This choice reflects current clinical practice and contemporary guideline recommendations, while allowing discussion of the biological heterogeneity that exists within each EF category. Rather than replacing the existing classification, the proposed conceptual framework is intended to complement it by providing a mechanistic interpretation of the phenotypes currently encountered in clinical practice.

The present review is based on the premise that understanding the organization of disease mechanisms may become as important as measuring ventricular function. Such an approach does not replace EF-based classification but complements it by providing a biologically informed framework that explains heterogeneity within existing categories.

### 1.2. Aims

This review proposes that differences between HFrEF and HFmrEF may reflect not only differences in left ventricular systolic function but also differences in the organization and dominance of underlying biological networks.

The specific objectives of this paper include the following: first, discussing the limitations of HF classification based solely on LVEF; second, presenting the key molecular and cellular mechanisms involved in the progression of HFrEF and HFmrEF; third, proposing a model in which therapeutic response depends on the position of the targeted pathway within the disease network; and fourth, linking biological mechanisms to clinical trial results, guideline recommendations, and phenotypically targeted treatment strategies.

The proposed framework is hypothetical and does not constitute a definitive classification system. Their aim is to integrate available mechanistic and clinical data and to provide direction for further research into more precise, mechanistically targeted classification and therapy of heart failure.

### 1.3. Literature Search and Conceptual Approach

The present manuscript was designed as a narrative, hypothesis-generating review aimed at developing a mechanistic framework for interpreting heart failure with reduced (HFrEF) and mildly reduced ejection fraction (HFmrEF) beyond conventional left ventricular ejection fraction (LVEF)-based classification. Rather than providing a systematic assessment of all available evidence, the objective was to integrate current knowledge from molecular biology, cardiovascular physiology, translational research, clinical trials, and contemporary guideline recommendations into a unified conceptual model.

The literature search primarily focused on publications from 2000 onwards, reflecting the period during which major advances in molecular cardiology, network biology, and evidence-based heart failure therapy have emerged. Seminal studies published before 2000 were also included, where necessary, to provide historical context and describe foundational pathophysiological mechanisms.

The literature search was performed using PubMed/MEDLINE, Scopus, and Web of Science, focusing on publications addressing the biological mechanisms underlying heart failure phenotypes, ventricular remodeling, neurohormonal activation, inflammation, endothelial dysfunction, mitochondrial biology, calcium handling, biomarkers, and contemporary pharmacological therapy. Search terms included combinations of heart failure, HFrEF, HFmrEF, HFimpEF, network biology, cardiac remodeling, neurohormonal activation, mitochondrial dysfunction, calcium handling, inflammation, biomarkers, clinical trials, and heart failure guidelines. Reference lists of selected publications were additionally screened to identify relevant studies that might not have been captured during the electronic search.

Priority was given to international heart failure guidelines, landmark randomized controlled trials, high-quality mechanistic studies, and influential review articles that have substantially contributed to the current understanding of heart failure biology. Earlier publications were included when they represented seminal work that established fundamental concepts relevant to contemporary heart failure research.

Evidence retrieved from the literature was not synthesized quantitatively. Instead, publications were critically evaluated for their relevance to the proposed conceptual framework. Particular emphasis was placed on studies illustrating interactions between molecular pathways, cellular processes, clinical phenotypes, therapeutic response, and disease progression. These data were subsequently integrated into a network-based interpretive model intended to explain the biological heterogeneity observed within conventional EF-defined heart failure categories.

The proposed framework should therefore be regarded as conceptual and hypothesis-generating, rather than a validated biological classification. Its purpose is to provide a mechanistic perspective that may facilitate interpretation of existing evidence, stimulate future translational research, and support the development of more biologically informed approaches to heart failure phenotyping and treatment.

## 2. Conceptual Framework: HF as a Network Disorder

HF may be viewed as a systems-level disorder in which multiple interacting molecular, cellular, and systemic processes determine disease progression and therapeutic response. Within this framework, the concept of network coherence explains the differences between HFrEF and HFmrEF [29,30].

**Figure 1:**
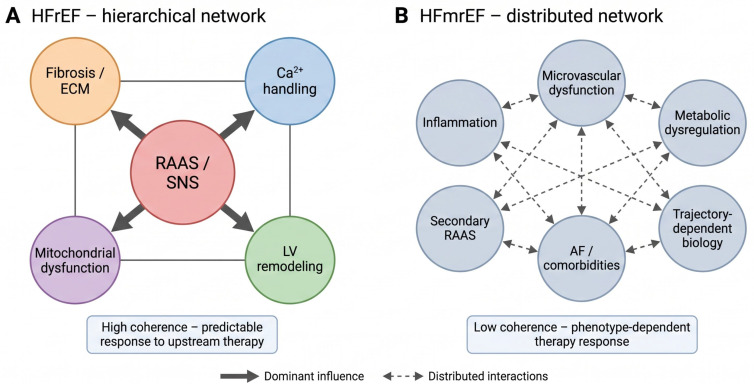
Network coherence in HFrEF and HFmrEF. Schematic comparison of HFrEF as a high-coherence, hierarchical network dominated by RAAS/SNS activation and HFmrEF as a low-coherence, distributed network shaped by multiple interacting mechanisms. Thick solid arrows indicate dominant influence, whereas dashed arrows indicate distributed interactions. AF—atrial fibrillation; Ca^2+^—calcium ions, ECM—extracellular matrix; HFmrEF—HF with mildly reduced ejection fraction; HFrEF—HF with reduced ejection fraction; LV—left ventricular; RAAS—renin–angiotensin–aldosterone system; SNS—sympathetic nervous system. Created in BioRender. Mertowski, S. (2026) https://BioRender.com/tc2ats7.

A key element of the proposed model is the concept of network coherence. In this paper, the term refers to the degree to which the disease course is controlled by a limited number of dominant, hierarchically organized pathways. In systems with high coherence, modulation of overarching regulatory mechanisms leads to relatively predictable biological and clinical outcomes. In contrast, systems with low coherence are characterized by distributed regulation, in which many partially active processes contribute to disease expression to varying degrees. It should be emphasized that network coherence is understood here as a conceptual construct for integrating mechanistic and clinical data, and not as a validated quantitative indicator (Figure 1).

From this perspective, HFrEF can be viewed as a phenotype with relatively greater pathophysiological coherence than HFmrEF in many patients, largely owing to the central role of neurohormonal activation. Nevertheless, HFrEF itself remains biologically heterogeneous and encompasses multiple underlying disease entities, including diverse forms of dilated cardiomyopathy with distinct genetic, inflammatory, ischemic, and toxic etiologies. Consequently, the concept of network coherence should be interpreted as a relative, rather than an absolute, characteristic of HFrEF biology [21,31,32].

If the proposed framework is correct, a relatively centralized neurohormonal network would be expected to confer greater sensitivity to therapies targeting these upstream regulatory pathways. The favorable results observed in randomized trials are therefore interpreted as consistent with this hypothesis rather than as evidence that the network is inherently centralized. HFmrEF represents a more heterogeneous category. In some patients, it reflects improvement from previous HFrEF; in others, early progression towards systolic dysfunction; and in another group, a phenotype similar to HFpEF, in which inflammation, endothelial dysfunction, microcirculatory disorders, metabolic dysregulation, and comorbidities play a more significant role [27,33,34].

Consequently, in HFmrEF, it is not always possible to identify a single dominant pathophysiological axis. The therapeutic response may therefore depend on which mechanism plays the most important role in a given patient.

The network model also allows for a different interpretation of HF pathophysiology. A therapeutic intervention can be viewed as an attempt to modify a specific node or pathway in the disease network. If the treatment target is central or limits the disease course, the clinical effect should be more pronounced and reproducible. At present, the centrality of individual pathways cannot be directly measured in routine clinical practice. Consequently, the proposed model should not be interpreted as inferring pathway dominance solely from treatment efficacy. Rather, it hypothesizes that independently measurable characteristics of biological network organization will ultimately explain differences in therapeutic responsiveness. However, if a given pathway is only one of many partially active mechanisms, the treatment response may be weaker, more variable, and dependent on the study population’s phenotype. This approach does not replace classification based on ejection fraction; rather, it complements it. LVEF remains an important clinical parameter but should be interpreted as the end result of a complex biological network rather than as a direct definition of disease mechanism. Incorporating a network perspective may therefore help us better understand the differences between HFrEF and HFmrEF, interpret variable clinical trial findings, and design more precise treatment strategies.

### Toward Operational Definitions of Network Coherence and Pathway Dominance

The concepts of *network coherence* and *pathway dominance* are introduced in this review as interpretive constructs intended to facilitate biological understanding of heart failure phenotypes rather than as currently established quantitative variables. Their purpose is to provide a systems-level framework for integrating molecular mechanisms, clinical phenotypes, and therapeutic responses. However, for such a framework to evolve beyond a conceptual model, these constructs must ultimately be translated into measurable biological features that can be examined experimentally.

From a systems biology perspective, network coherence may be understood as the degree to which disease behavior is governed by a limited number of highly interconnected regulatory pathways whose activity influences multiple downstream biological processes. In a highly coherent network, modulation of one or a few central pathways would be expected to produce coordinated changes across numerous molecular and cellular mechanisms, resulting in relatively predictable biological and clinical responses. Conversely, a network with low coherence would be characterized by distributed regulation involving several partially independent processes, so that inhibition of a single pathway would be expected to produce more variable therapeutic effects.

Within this framework, pathway dominance should not be interpreted simply as the magnitude of activation of an individual signaling cascade. Rather, it refers to the relative influence that a biological pathway exerts over the organization and behavior of the overall disease network. A pathway may therefore be considered dominant when it occupies a central regulatory position, coordinating multiple downstream mechanisms involved in myocardial remodeling, inflammation, metabolism, vascular dysfunction, or neurohormonal activation. Importantly, pathway dominance is conceived as a property of network architecture rather than isolated molecular abundance.

Although no validated clinical metric currently exists for either construct, contemporary network biology provides several methodological approaches that could facilitate their future operationalization. Patient-specific transcriptomic, proteomic, metabolomic, or multimodal datasets may be used to reconstruct molecular interaction networks, allowing quantitative characterization of network organization. Potential analytical approaches include weighted gene co-expression network analysis (WGCNA), Bayesian network inference, protein–protein interaction network analysis, causal network reconstruction, and graph-based machine learning methods. Although no validated clinical metric currently exists, future studies could operationalize network coherence by reconstructing patient-specific networks from transcriptomic, proteomic, metabolomic, or multimodal datasets. Network topology measures—including node centrality, modularity, clustering coefficient, and entropy—could then serve as candidate quantitative descriptors of network organization. Within these reconstructed networks, measures such as node centrality, modularity, network entropy, clustering coefficients, or pathway enrichment scores could provide complementary descriptions of the degree of network organization and the relative influence of individual biological pathways.

Importantly, the present framework does not propose any single metric as a definitive measure of network coherence. Rather, we hypothesize that different computational approaches may converge on a common biological principle: patients whose disease networks exhibit greater centralization around a limited number of regulatory hubs may demonstrate more consistent responses to therapies targeting those hubs than patients whose disease biology is distributed across multiple interacting mechanisms. The identification of robust quantitative measures to test this hypothesis remains an important objective for future translational research.

Accordingly, the therapeutic observations discussed throughout this review should not be interpreted as evidence establishing the existence of network coherence or pathway dominance. Instead, the reproducible clinical benefits observed with neurohormonal blockade in HFrEF are considered compatible with the proposed network architecture. Conversely, the greater variability of treatment responses observed in HFmrEF is interpreted as being consistent with a more distributed biological organization. These clinical observations therefore motivate rather than prove the proposed conceptual model.

This perspective generates several testable hypotheses. First, patient-specific measures of network organization derived from multi-omics analyses may correlate more closely with therapeutic responsiveness than LVEF alone. Second, individuals demonstrating greater centralization of neurohormonal signaling may derive larger benefits from neurohormonal inhibition than patients with biologically distributed inflammatory or metabolic phenotypes. Finally, longitudinal changes in network architecture may accompany transitions between heart failure phenotypes, providing mechanistic insight into disease progression and therapeutic remodeling. These hypotheses require prospective validation but illustrate how the proposed conceptual framework can be transformed into an experimentally testable research program.

The proposed framework is inherently falsifiable. It would be challenged if future network-based analyses demonstrate that quantitative measures of pathway centrality or network organization do not correlate with therapeutic responsiveness more closely than conventional LVEF-based classification. Conversely, demonstration of such associations would provide empirical support for the biological relevance of the proposed conceptual model.

## 3. Mechanistic Architecture of Heart Failure

### 3.1. Neurohormonal and Systemic Mechanisms

**Figure 2:**
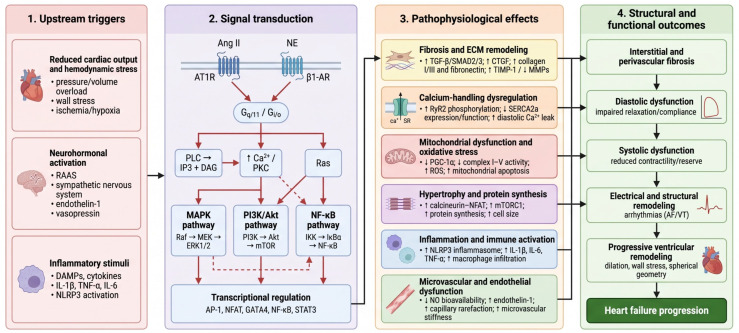
Molecular signaling cascade in heart failure. Schematic overview of the main upstream triggers, intracellular signaling pathways, pathophysiological effects, and structural–functional consequences involved in HF progression. Ang II—angiotensin II; AT1R—angiotensin II type 1 receptor; β1-AR—β1-adrenergic receptor; CTGF—connective tissue growth factor; DAG—diacylglycerol; DAMPs—damage-associated molecular patterns; ECM—extracellular matrix; ERK—extracellular signal-regulated kinase; GATA4—GATA-binding protein 4; Gi/o—Gi/o heterotrimeric G protein, Gq/11—Gq/11 heterotrimeric G protein; IκBα—inhibitor of nuclear factor kappa B alpha; IKK—IκB kinase, IL—interleukin; IP3—inositol trisphosphate; MAPK—mitogen-activated protein kinase; MEK—mitogen-activated protein kinase kinase, MMPs—matrix metalloproteinases; mTOR—mechanistic target of rapamycin; NE—norepinephrine; NFAT—nuclear factor of activated T cells; NF-κB—nuclear factor kappa B; NLRP3—NOD-like receptor family pyrin domain containing 3; NO—nitric oxide; PGC-1α—peroxisome proliferator-activated receptor gamma coactivator 1-alpha; PI3K/Akt—phosphoinositide 3-kinase/protein kinase B; PKC—protein kinase C; PLC—phospholipase C; RAAS—renin–angiotensin–aldosterone system; Raf—rapidly accelerated fibrosarcoma kinase (RAF kinase), Ras—Ras small GTPase, ROS—reactive oxygen species; RyR2—ryanodine receptor 2; SERCA2a—sarcoplasmic/endoplasmic reticulum Ca^2+^-ATPase 2a; SNS—sympathetic nervous system; STAT3—signal transducer and activator of transcription 3; TGF-β—transforming growth factor beta; TIMP—tissue inhibitor of metalloproteinase; TNF-α—tumor necrosis factor alpha. Arrow notation: Solid red arrows indicate primary activation of intracellular signaling pathways. Dashed red arrows indicate pathway cross-talk, secondary regulation, or feedback interactions. Solid black arrows indicate downstream biological progression from upstream mechanisms to pathophysiological effects and ultimately to structural and functional consequences. **↑** indicates increased expression, activation, or activity. **↓** indicates decreased expression, inhibition, or impaired function. Created in BioRender. Mertowski, S. (2026) https://BioRender.com/tc2ats7.

LVEF is one of the most important clinical parameters used to classify HF, but its value reflects the end result of many interrelated biological processes. LVEF depends not only on cardiomyocyte contractility but also on left ventricular geometry, wall compliance, preload and afterload, valvular function, heart rate, and vascular status. Therefore, it should not be considered a direct marker of a specific disease mechanism, but rather as an outcome parameter resulting from complex interactions between the myocardium, vascular system, kidneys, and neurohormonal systems. In HFrEF, one of the best-understood and most consistently documented mechanisms of disease progression is the chronic activation of the renin–angiotensin–aldosterone system (RAAS) and the sympathetic nervous system (SNS) (Figure 2) [35,36,37,38,39].

Initially, activation of these systems is compensatory. Reduced cardiac output activates mechanisms designed to maintain organ perfusion, blood pressure, and circulating blood volume. In the short term, increased sympathetic activity, sodium and water retention, and increased vascular tone can stabilize circulation. However, in chronic conditions, these same mechanisms become maladaptive and contribute to further myocardial damage.

Angiotensin II and aldosterone enhance vasoconstriction, sodium retention, fibroblast activation, oxidative stress, and extracellular matrix deposition. Concurrently, chronic adrenergic stimulation increases heart rate, myocardial oxygen demand, susceptibility to arrhythmias, and catecholamine toxicity to cardiomyocytes. The combined action of the RAAS and SNS leads to increased hemodynamic load, cardiomyocyte hypertrophy, fibrosis, left ventricular dilatation, and a gradual loss of contractile reserve. In this sense, neurohormonal activation is not merely a response to HF but rather one of the central mechanisms that maintain and drive its progression [40,41].

Mechanotransduction, the conversion of mechanical stimuli into biochemical responses, is also crucial. Increased left ventricular wall stress, volume or pressure overload, and altered extracellular matrix stiffness activate pathways dependent on integrins, adhesion kinases, cytoskeletal proteins, and sarcomeric structures, including titin [42].

These mechanisms enable myocardial cells to sense mechanical stress and translate it into a molecular response. However, under conditions of chronic overload, this response shifts toward maladaptive hypertrophy, cytoskeletal remodeling, fibroblast activation, and impaired ventricular mechanical properties.

At this level, the network nature of HF becomes apparent. Activation of the RAAS and SNS, hemodynamic overload, mechanotransduction, oxidative stress, and fibrosis are not discrete, linear phenomena. Rather, they form a system of interconnected processes, in which one process amplifies the others. Wall overload activates hypertrophic pathways; hypertrophy and fibrosis increase ventricular stiffness; this stiffness worsens filling conditions and increases intracardiac pressures, which in turn enhances neurohormonal activation. In many patients with HFrEF, the available mechanistic evidence suggests that neurohormonal activation occupies an upstream regulatory position within the broader pathophysiological network. Within the present conceptual framework, this observation forms the basis for the hypothesis that HFrEF frequently exhibits a relatively centralized network architecture, although this proposition awaits direct experimental validation.

### 3.2. Intracellular Signaling and Myocardial Remodeling

Neurohormonal activation and mechanical overload are translated into a cellular response via numerous signaling cascades. Angiotensin II, acting primarily through the angiotensin II type 1 receptor (AT1), activates mitogen-activated protein kinase (MAPK) pathways, including ERK1/2, JNK, and p38 MAPK. These pathways regulate the expression of genes associated with cardiomyocyte hypertrophy, fibroblast proliferation, cytokine production, and extracellular matrix remodeling. Their chronic activation promotes a shift in the adaptive response toward pathological remodeling [43,44,45].

Concurrently, activation of NADPH oxidases leads to increased production of reactive oxygen species (ROS). ROS act as signaling molecules, but their excess leads to oxidative stress, damage to proteins, lipids, and DNA, and the activation of transcription factors such as NF-κB and AP-1 [46,47,48].

In this way, oxidative stress amplifies the inflammatory response, enhances fibroblast activation, and perpetuates the maladaptive remodeling program. Importantly, oxidative stress not only results from neurohormonal activation but also, in turn, exacerbates mitochondrial dysfunction, calcium metabolism disorders, and endothelial damage [49,50].

A particularly important element of remodeling is the activation of the transforming growth factor beta (TGF-β) pathway and SMAD proteins [51,52]. The TGF-β/SMAD pathway promotes fibroblast differentiation into myofibroblasts, increased collagen synthesis, and extracellular matrix deposition. In the short term, fibrosis may stabilize the structure of damaged myocardium. In the long term, however, it leads to increased wall stiffness, impaired relaxation, impaired electrical conduction, and an increased risk of arrhythmia [53,54].

Myocardial remodeling involves not only cardiomyocytes but also fibroblasts, endothelial cells, vascular smooth muscle cells, macrophages, and other immune cells. HF is therefore a multicellular process. Cardiomyocytes respond to overload with hypertrophy, metabolic disruption, and altered gene expression; fibroblasts regulate the composition and stiffness of the extracellular matrix; endothelial cells influence perfusion, nitric oxide bioavailability, and vascular tone; and immune cells modulate inflammation and repair processes. The interactions between these cell populations are one of the main sources of phenotypic heterogeneity in HF [55,56].

Chronic β-adrenergic stimulation additionally leads to desensitization and internalization of β-adrenergic receptors, inter alia, via G protein-coupled receptor kinases, particularly GRK2. This results in reduced efficiency of cyclic adenosine monophosphate (cAMP)-dependent signaling, reduced contractile reserve, and impaired phosphorylation of proteins responsible for excitation–contraction coupling. This mechanism constitutes an important link between chronic neurohormonal activation, contractile dysfunction, and calcium metabolism disorders [57,58,59].

### 3.3. Calcium Disturbances and Excitation–Contraction Coupling

Normal contractile function of the cardiac muscle depends on a precisely regulated calcium cycle. Under physiological conditions, depolarization of the cell membrane leads to a small influx of Ca^2+^ through L-type calcium channels, which induces the release of more calcium from the sarcoplasmic reticulum via the ryanodine receptor 2 (RyR2). The increased calcium concentration in the cytoplasm enables the interaction of actin and myosin and cardiomyocyte contraction. Calcium is then either reuptaken into the sarcoplasmic reticulum by SERCA2a or removed from the cell, enabling relaxation [60].

In HF, this mechanism is disrupted at multiple levels. Reduced expression and activity of SERCA2a limit calcium reuptake into the sarcoplasmic reticulum, while post-translational modifications of RyR2, including hyperphosphorylation and oxidation, promote diastolic calcium leak. Consequently, there is reduced calcium available for contraction, prolonged relaxation, increased cytoplasmic calcium concentration during diastole, and increased susceptibility to arrhythmias. These disturbances therefore affect both systolic and diastolic function [61,62,63].

Calcium metabolism is also closely linked to β-adrenergic signaling. Under normal conditions, stimulation of β-adrenergic receptors increases cAMP availability and protein kinase A activity, thereby enhancing the phosphorylation of proteins involved in excitation–contraction coupling and increasing contractile force. In chronic heart failure, this mechanism is impaired by receptor desensitization, altered kinase and phosphatase activity, and structural abnormalities of the calcium apparatus. As a result, the heart muscle loses its ability to adequately increase contractility in response to hemodynamic stress or exercise [64,65,66].

From a network perspective, calcium metabolism disorders constitute a convergence point of many upstream pathological processes. Neurohormonal activation, oxidative stress, mitochondrial dysfunction, and structural remodeling affect proteins that regulate the calcium cycle, and a disturbed calcium cycle, in turn, exacerbates metabolic dysfunction, arrhythmogenesis, and cellular damage. Therefore, calcium metabolism can be considered one of the key intermediary nodes between molecular signaling and the clinical phenotype of heart failure.

### 3.4. Mitochondrial Dysfunction and Metabolic Stress

The heart muscle is an organ with very high energy demands, and the proper functioning of cardiomyocytes depends on the continuous production of ATP, primarily in the mitochondria. HF leads to impaired energy metabolism, reduced efficiency of oxidative phosphorylation, increased production of reactive oxygen species, and reduced metabolic flexibility. This means that cardiomyocytes lose the ability to effectively adapt their energy substrate utilization to changing hemodynamic and metabolic conditions.

**Figure 3:**
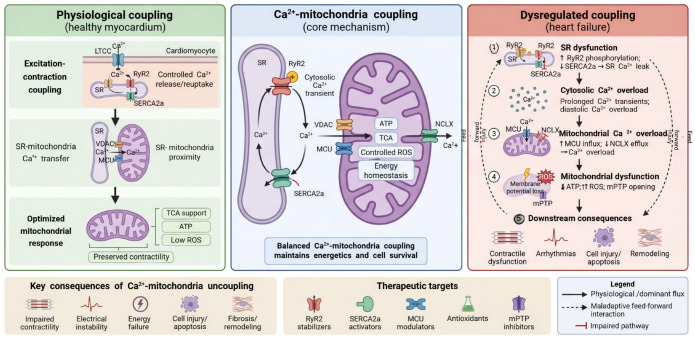
Calcium–mitochondria coupling in heart failure. Schematic representation of physiological and dysregulated Ca^2+^–mitochondria coupling in cardiomyocytes. The figure illustrates how impaired SR Ca^2+^ handling, mitochondrial Ca^2+^ overload, reduced ATP production, increased ROS generation, and mPTP opening contribute to contractile dysfunction, electrical instability, cell injury, apoptosis, fibrosis, and adverse remodeling. Arrow notation: Solid black arrows indicate physiological calcium transport, dominant intracellular signaling, or principal downstream biological effects. Dashed black arrows indicate maladaptive feed-forward interactions and reciprocal amplification between calcium dysregulation and mitochondrial dysfunction. Red inhibitory symbols (⊣) indicate impaired biological processes or reduced functional activity. Upward arrows (↑) indicate increased activity, overload, or activation, whereas downward arrows (**↓**) indicate decreased expression, impaired function, or reduced activity. Abbreviations: ATP—adenosine triphosphate; Ca^2+^—calcium ion; ΔΨm—mitochondrial membrane potential; LTCC—L-type calcium channel; MCU—mitochondrial calcium uniporter; mPTP—mitochondrial permeability transition pore; NCLX—mitochondrial sodium/calcium exchanger; RIRR—ROS-induced reactive oxygen species release; ROS—reactive oxygen species; RyR2—ryanodine receptor 2; SERCA2a—sarcoplasmic/endoplasmic reticulum Ca^2+^-ATPase 2a; SR—sarcoplasmic reticulum; TCA—tricarboxylic acid cycle; VDAC—voltage-dependent anion channel. Created in BioRender. Mertowski, S. (2026) https://BioRender.com/tc2ats7.

Mitochondrial dysfunction is closely linked to impaired calcium metabolism (Figure 3). Under physiological conditions, transient calcium uptake by mitochondria stimulates enzymes of the tricarboxylic acid cycle and supports ATP production. In heart failure, chronically elevated cytoplasmic calcium concentration can lead to mitochondrial calcium overload, loss of mitochondrial membrane potential, opening of the mitochondrial permeability transition pore, impaired ATP production, and increased oxidative stress. In turn, excess ROS damages calcium channel proteins, RyR2 and SERCA2a, further exacerbating calcium cycling disorders.

This creates a positive feedback loop in which mitochondrial dysfunction and calcium metabolism disorders mutually reinforce each other. ATP deficiency impairs the function of ion pumps and the contractile apparatus, thereby limiting systolic and diastolic function. Simultaneously, oxidative stress activates inflammatory and profibrotic pathways, intensifying structural remodeling. This mechanism links bioenergetic disturbances with cell damage, arrhythmogenesis, and progression of HF.

The importance of metabolic dysfunction is particularly evident in phenotypes characterized by obesity, insulin resistance, diabetes, chronic kidney disease, and chronic inflammation. Under such conditions, impaired energy substrate utilization, lipotoxicity, oxidative stress, and microvascular dysfunction can significantly affect the disease phenotype, even in the presence of a relatively preserved or only mildly reduced ejection fraction. This is particularly relevant to the interpretation of HFmrEF as a category encompassing both HFrEF-like and HFpEF-like phenotypes.

### 3.5. Inflammation, Endothelium, and Microcirculation

In addition to classic neurohormonal mechanisms, inflammation, endothelial dysfunction, and microcirculation disorders are gaining increasing importance. These processes are particularly important in HFpEF-like phenotypes and in a subset of the HFmrEF population [67,68,69,70].

Chronic, low-grade inflammation, associated with conditions such as obesity, diabetes, aging, kidney disease, and hypertension, leads to endothelial cell activation, increased cytokine production, oxidative stress, and impaired nitric oxide bioavailability [71,72].

Reduced nitric oxide bioavailability limits the activation of the cGMP–PKG pathway, which may promote titin hypophosphorylation and increased cardiomyocyte stiffness. As a result, impaired relaxation and increased myocardial stiffness, rather than a primary loss of contractility, may be the dominant problem. This mechanism is particularly characteristic of HFpEF but may also play a significant role in some patients with HFmrEF [73,74].

Activation of innate immunity, including pathways associated with the NLRP3 inflammasome, may further enhance proinflammatory cytokine production, fibroblast activation, and endothelial dysfunction [75,76,77].

Unlike classic HFrEF, HFpEF-like biology is often characterized by distributed regulation involving multiple interacting systemic mechanisms, which may contribute to greater variability in therapeutic response.

### 3.6. The Importance of Mechanistic Architecture in the Differences Between HFrEF and HFmrEF

The mechanisms described indicate that the differences between HFrEF and HFmrEF are not solely due to differences in ejection fraction but rather to differences in the organization of biological processes. In HFrEF, neurohormonal activation, structural remodeling, abnormalities in calcium metabolism, and mitochondrial dysfunction often form a relatively coherent, mutually reinforcing system. Predominant, overarching pathways control multiple secondary processes; therefore, their pharmacological modulation can lead to broad and reproducible clinical effects [78].

This interpretation provides the basis for further discussion of HFmrEF phenotypes and for proposals for treatment that targets not only LVEF but also the dominant pathophysiological mechanisms.

## 4. HFmrEF as a Heterogeneous Category

### 4.1. The Position of HFmrEF in the HF Spectrum

HFmrEF occupies an intermediate position in the LVEF-based classification, typically ranging from 41% to 49%. However, the mere position between HFrEF and HFpEF does not imply the existence of a distinct, homogeneous biological entity. HFmrEF should rather be considered a transitional, multi-mechanistic category, in which similar LVEF values may result from different disease trajectories and distinct pathophysiological configurations [79,80]. From a network perspective, HFmrEF encompasses multiple biological trajectories that differ in their dominant molecular mechanisms and disease evolution [21,81,82]. These trajectories are described below.

It should also be acknowledged that the clinical relevance of HFmrEF as a separate heart failure phenotype remains a matter of ongoing debate. Several investigators have questioned whether this category represents a biologically distinct entity or merely reflects a transitional stage between HFrEF and HFpEF [83,84,85]. More recently, it has been proposed that future heart failure classification should move beyond rigid EF thresholds and instead incorporate disease trajectory, imaging characteristics, biomarkers, and underlying biological mechanisms. Within this context, the present review does not seek to reinforce HFmrEF as a fixed disease category but rather uses it as a clinically recognized framework for discussing the biological diversity currently grouped within this EF range. Our proposed framework is therefore aligned with recent calls to move beyond rigid EF thresholds toward a more biologically informed approach to heart failure phenotyping [86].

Consequently, EF in HFmrEF does not define a single disease mechanism but rather indicates an area of overlap between different biological phenotypes. This heterogeneity has direct clinical significance. Patients classified in the same EF category may differ in terms of their medical history, left ventricular structure, biomarker profile, degree of fibrosis, presence of ischemia, heart rhythm, metabolic diseases, and response to treatment. Therefore, the interpretation of HFmrEF requires taking into account not only the current LVEF value but also the previous EF, the dynamics of change over time, the structural phenotype, comorbidities, and markers of activity in individual pathophysiological pathways.

### 4.2. Main Biological Phenotypes of HFmrEF

Building on this biological heterogeneity, three major phenotypic trajectories can be distinguished. These are not entirely distinct, but they help to link the clinical picture to the predominant disease mechanisms and potential therapeutic response [34].

The first phenotype comprises patients with heart failure with improved ejection fraction (HFimpEF), defined by current guidelines as individuals with a prior LVEF ≤40% who subsequently achieve an LVEF > 40% following treatment or recovery. In this group, the preexisting systolic dysfunction partially improved with treatment or modification of the causative factor. Despite an increase in LVEF, complete resolution of the disease should not be assumed. Many patients may experience residual neurohormonal abnormalities, fibrosis, extracellular matrix remodeling, altered β-adrenergic sensitivity, and incomplete normalization of calcium [87]. This phenotype can therefore be interpreted as a state of remission or partial stabilization, in which the disease network remains susceptible to reactivation, particularly after treatment discontinuation.

The second phenotype is HFmrEF, characterized by early or progressive systolic dysfunction. In these patients, the current LVEF value may precede the development of full-blown HFrEF. Mechanistically, increasing activation of the renin–angiotensin–aldosterone system (RAAS) and the sympathetic nervous system (SNS), early left ventricular remodeling, initial calcium cycling abnormalities, mitochondrial dysfunction, and increased oxidative stress are observed [88].

Unlike persistent HFrEF, these processes may not yet be fully consolidated, creating a potential window for early therapeutic intervention. Recognizing this phenotype is particularly important because intensifying treatment targeting the mechanisms underlying HFrEF may limit further progression.

The third phenotype encompasses HFmrEF, which is biologically similar to HFpEF. Systemic and vascular factors, such as hypertension, obesity, insulin resistance, diabetes, chronic kidney disease, atrial fibrillation, chronic inflammation, and microcirculatory dysfunction, may play a central role in this group [89].

Impaired nitric oxide bioavailability, impaired cyclic guanosine monophosphate–protein kinase G (cGMP-PKG)-dependent signaling, oxidative stress, and inflammatory activation may lead to increased cardiomyocyte stiffness, impaired relaxation, and diastolic dysfunction. In this setting, EF remains only mildly reduced because the primary problem may not be a loss of contractility per se, but rather a complex interaction among myocardial stiffness, endothelial dysfunction, hemodynamic overload, and comorbidities.

The key differences between these three phenotypes, their typical clinical trajectories, dominant mechanisms, and therapeutic implications are summarized in Table 1 [90,91,92].

**Figure 4:**
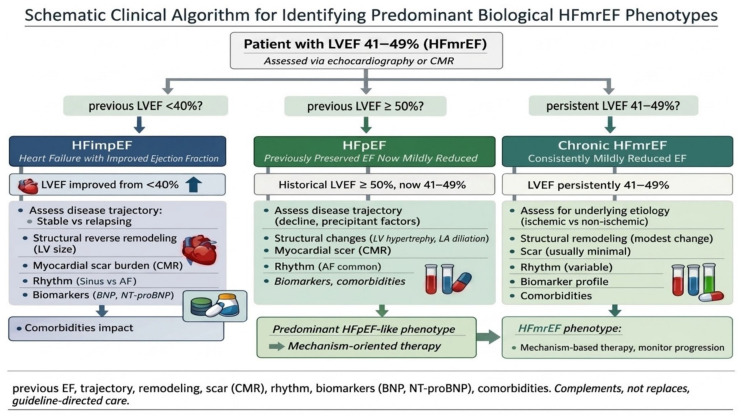
Schematic clinical algorithm for identifying predominant phenotypes. Created with www.figurelabs.ai by T. Urbanowicz (9 July 2026) ID: FL-PUB-20260709-VBVYXA. Abbreviations: AF, atrial fibrillation; BNP, B-type natriuretic peptide; CMR, cardiac magnetic resonance; EF, ejection fraction; HF, heart failure; HFimpEF, heart failure with improved ejection fraction; HFmrEF, heart failure with mildly reduced ejection fraction; HFpEF, heart failure with preserved ejection fraction; LA, left atrial; LV, left ventricular; LVEF, left ventricular ejection fraction; NT-proBNP, N-terminal pro-B-type natriuretic peptide.

While the biological phenotypes summarized above provide a conceptual framework for understanding HFmrEF heterogeneity, their clinical application requires integration of disease trajectory with structural, functional, and biological information. Rather than relying solely on the current ejection fraction, assessment should incorporate previous LVEF, ventricular remodeling, myocardial scar burden, cardiac rhythm, imaging findings, biomarkers, and major comorbidities. The proposed algorithm (Figure 4) illustrates a possible stepwise approach to interpreting HFmrEF from a biologically informed perspective, complementing contemporary guideline-based evaluation.

### 4.3. Implications of HFmrEF Heterogeneity for the Interpretation of Clinical Trials

Recognition of the biological diversity within HFmrEF provides important context for interpreting the heterogeneous outcomes observed across clinical trials. If the study population includes patients with different disease trajectories and different predominant mechanisms, the treatment effect may be blurred. Treatments targeting the neurohormonal mechanisms of HFrEF may be more beneficial in patients with preexisting low EF, left ventricular dilatation, high natriuretic peptide levels, ischemic cardiomyopathy, or progressive systolic dysfunction. However, the same drug may have a weaker effect in a population with an HFpEF-like phenotype characterized by inflammatory, metabolic, endothelial, or comorbidity-related processes [27,34].

These observations suggest that inconsistent treatment effects across HFmrEF populations may reflect biological heterogeneity rather than true therapeutic inefficacy. In a network model, therapeutic response depends on whether the drug’s mechanism of action aligns with the dominant disease configuration. If this is the case, the clinical effect may be significant; if not, the response will be limited or difficult to detect in the entire, biologically mixed population [93].

Sodium–glucose cotransporter 2 (SGLT2) inhibitors occupy a special place, as their actions span multiple biological domains, including volume regulation, renal function, metabolism, inflammation, and myocardial energetics [94,95].

Their broad, multifaceted profile of action may partially explain their more consistent benefits across a broader spectrum of HF, including patients with HFmrEF. However, this does not mean that HFmrEF is a therapeutically uniform category; rather, some interventions may target more conserved, systemic axes of HF biology [96,97].

### 4.4. Implications for Clinical Strategy

**Figure 5:**
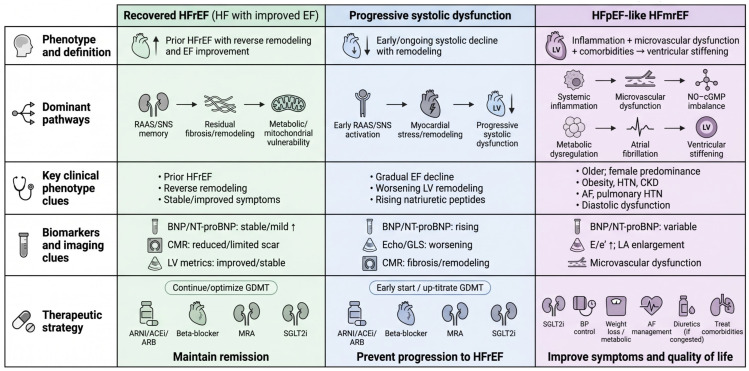
Phenotype-driven therapeutic framework for HFmrEF [78,82,83]. Proposed framework linking major HFmrEF phenotypes with dominant biological pathways, clinical phenotype clues, biomarker and imaging features, and corresponding therapeutic strategies. Colored panels represent distinct biological HFmrEF phenotypes and do not imply rigid diagnostic categories; overlap between phenotypes may occur in individual patients. ACEi—angiotensin-converting enzyme inhibitors; AF—atrial fibrillation; ARB—angiotensin receptor blockers; ARNI—angiotensin receptor–neprilysin inhibitor; BNP—B-type natriuretic peptide; BP—blood pressure; CKD—chronic kidney disease; CMR—cardiac magnetic resonance; EF—ejection fraction; E/e′—ratio of early transmitral flow velocity to early diastolic mitral annular velocity; GDMT—guideline-directed medical therapy; GLS—global longitudinal strain; HFmrEF—HF with mildly reduced ejection fraction; HFpEF—HF with preserved ejection fraction; HFrEF—HF with reduced ejection fraction; HTN—hypertension; LA—left atrial; LV—left ventricular; LVEF—left ventricular ejection fraction; MRA—mineralocorticoid receptor antagonist; NO–cGMP—nitric oxide–cyclic guanosine monophosphate; NT-proBNP—N-terminal pro-B-type natriuretic peptide; RAAS—renin–angiotensin–aldosterone system; SGLT2i—sodium–glucose cotransporter 2 inhibitor; SNS—sympathetic nervous system. Arrow notation: Solid black arrows indicate the principal clinical workflow, linking disease trajectory and biological phenotype with dominant pathophysiological mechanisms, diagnostic evaluation, and phenotype-directed therapeutic strategies. Dashed black arrows indicate overlapping biological mechanisms, shared pathophysiological pathways, or alternative phenotype transitions that may influence therapeutic decision-making. Converging arrows indicate integration of multiple clinical, imaging, and biomarker findings into biological phenotyping and individualized treatment selection. Bidirectional arrows (↔) indicate reciprocal interactions between biological mechanisms and clinical phenotype, emphasizing that disease progression and therapeutic response continuously influence one another. Upward arrows (↑) indicate increased activation, severity, or predominance of a biological process or clinical feature. Downward arrows (↓) indicate reduced activity, attenuation, improvement, or suppression of a biological process or clinical feature. Created in BioRender. Mertowski, S. (2026) https://BioRender.com/tc2ats7.

From a practical perspective, the evaluation of patients with HFmrEF should extend beyond the current LVEF value. Although ejection fraction remains an essential component of heart failure classification, therapeutic decisions should also consider the patient’s disease trajectory, structural cardiac phenotype, rhythm status, burden of myocardial fibrosis or scar, biomarker profile, and major comorbidities. Such an integrated assessment may facilitate identification of the predominant biological phenotype and improve selection of appropriate treatment strategies (Figure 5).

This approach does not negate the usefulness of LVEF-based classification but rather indicates that EF should be interpreted within a broader biological and clinical context. HFmrEF is a particularly good example of a situation in which the numerically defined EF category encompasses diverse disease mechanisms. Therefore, future classification and treatment models should integrate LVEF with data on disease trajectory, imaging, biomarkers, comorbidities, and dominant pathophysiological pathways.

### 4.5. A Practical Phenotype-Oriented Approach to HFmrEF

Because HFmrEF encompasses biologically heterogeneous patient populations, a stepwise clinical assessment may help place an individual patient within the proposed conceptual framework.

Step 1. Determine the LVEF trajectory.

Previous echocardiographic examinations should be reviewed to establish whether HFmrEF represents recovery from previous HFrEF (HFimpEF), progressive deterioration of systolic function, or a stable mildly reduced ejection fraction.

Step 2. Assess structural cardiac phenotype.

Left ventricular dimensions, remodeling pattern, hypertrophy, left atrial enlargement, diastolic function, and myocardial scar or fibrosis identified by cardiac magnetic resonance imaging may provide important information regarding the underlying disease process.

Step 3. Evaluate cardiac rhythm.

The presence of atrial fibrillation, ventricular arrhythmias, or conduction abnormalities should be considered, as rhythm disturbances substantially influence symptoms, remodeling, biomarker concentrations, and treatment strategies.

Step 4. Integrate biomarkers with imaging findings.

Natriuretic peptides, cardiac troponins, and selected inflammatory or fibrosis biomarkers may provide complementary information regarding disease activity. However, these biomarkers should always be interpreted within the broader clinical context because they do not directly identify the dominant biological pathway.

Step 5. Identify major comorbidities.

Obesity, diabetes mellitus, chronic kidney disease, hypertension, ischemic heart disease, and other systemic disorders frequently shape the biological phenotype of HFmrEF and may substantially influence therapeutic response.

Step 6. Tailor treatment according to the predominant phenotype while maintaining guideline-directed medical therapy whenever appropriate.

Patients with features suggestive of persistent HFrEF biology may derive greater benefit from comprehensive neurohormonal blockade, whereas patients with an HFpEF-like phenotype may require greater emphasis on management of comorbidities, congestion, metabolic dysfunction, and systemic inflammation.

**Figure 6:**
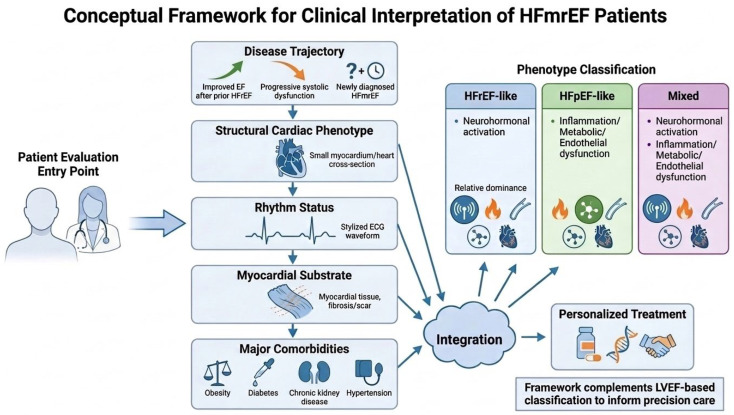
Stepwise biological interpretation of heart failure with mildly reduced ejection fraction (HFmrEF). Proposed conceptual framework for the clinical interpretation of patients with HFmrEF. Rather than considering HFmrEF as a single biological entity, the framework emphasizes sequential evaluation of disease trajectory, structural cardiac phenotype, rhythm status, myocardial substrate, and major comorbidities to identify the predominant pathophysiological mechanisms. The initial assessment distinguishes patients with improved ejection fraction following previous HFrEF, progressive systolic dysfunction, or newly diagnosed HFmrEF without a documented disease trajectory. Subsequent evaluation integrates imaging findings, myocardial fibrosis or scar burden, atrial fibrillation, and systemic comorbidities, including obesity, diabetes mellitus, chronic kidney disease, and hypertension. This stepwise approach facilitates classification into HFrEF-like, HFpEF-like, or mixed biological phenotypes, which may differ in the relative contribution of neurohormonal activation, inflammation, endothelial dysfunction, metabolic dysregulation, and myocardial remodeling. The proposed framework is intended as a hypothesis-generating conceptual model that complements conventional LVEF-based classification and illustrates how biological interpretation may ultimately support more individualized therapeutic strategies as future mechanistic biomarkers and network-based approaches become clinically available. Abbreviations: AF—atrial fibrillation; CKD—chronic kidney disease; EF—ejection fraction; HF—heart failure; HFmrEF—heart failure with mildly reduced ejection fraction; HFpEF—heart failure with preserved ejection fraction; HFrEF—heart failure with reduced ejection fraction; LVEF—left ventricular ejection fraction. Created by FigureLabs by Urbanowicz T. (ID: FL-PUB-20260626-U1I5WM).

The application of the proposed framework in clinical practice requires a structured interpretation that integrates disease trajectory, imaging findings, and the burden of accompanying comorbidities. Figure 6 summarizes a proposed stepwise approach for the biological interpretation of HFmrEF, illustrating how conventional EF assessment may be complemented by sequential evaluation of mechanistic features that could influence therapeutic decision-making.

## 5. Therapeutic Response as a Function of Pathway Dominance

The effectiveness of HF treatment depends not only on the drug class itself but also on whether the therapy’s mechanism of action aligns with the dominant biological processes in a given patient. In a network approach, a drug can be considered an intervention that modifies a specific node or pathway within the disease network. If the therapeutic target is central, overriding, or limits disease progression, the clinical effect should be more pronounced and reproducible. However, if a given pathway is only one of many partially active mechanisms, the response may be weaker, more variable, and phenotype-dependent [98,99,100,101].

In HFrEF, many basic therapies target pathways that play a key role in maintaining the maladaptive disease network. Angiotensin-converting enzyme inhibitors, angiotensin receptor blockers, angiotensin receptor and neprilysin inhibitors, β-blockers, and mineralocorticoid receptor antagonists limit activation of the renin–angiotensin–aldosterone system (RAAS), adrenergic signaling, sodium retention, fibrosis, oxidative stress, and left ventricular remodeling. Because these processes are tightly coupled in many patients with HFrEF and occupy a central place in the disease architecture, modulating them has effects beyond a single drug target. Therefore, HFrEF treatment can be interpreted as an example of effective modulation of a system with relatively high network coherence. Blockade of overarching neurohormonal pathways influences numerous secondary processes, including fibrosis, cardiomyocyte hypertrophy, calcium metabolism disorders, mitochondrial dysfunction, and progressive ventricular remodeling [102]. This explains why therapies with different direct targets can collectively reduce mortality, hospitalization, and disease progression. In this sense, treatment benefits stem not only from inhibiting a single mechanism but also from interrupting mutually reinforcing pathophysiological loops. In HFmrEF, the relationship between the mechanism of drug action and clinical response is more complex. If HFmrEF represents improvement from previous HFrEF or the early phase of progression to HFrEF, neurohormonal pathways may still play a significant role in the disease network [39]. In such cases, therapies specific to HFrEF may be more beneficial, especially in patients with preexisting low EF, left ventricular dilatation, ischemic cardiomyopathy, elevated natriuretic peptide levels, or signs of active remodeling [103,104,105].

However, in patients with HFmrEF with an HFpEF-like phenotype, inflammatory, metabolic, endothelial, or comorbidity-related mechanisms may predominate. In this situation, blocking classic neurohormonal pathways may have a more limited effect. SGLT2 inhibitors are particularly important in this context. Their actions encompass multiple biological domains, including intravascular volume regulation, renal function, sodium metabolism, energy metabolism, oxidative stress, and inflammatory signaling [106,107].

Unlike therapies that primarily target a single neurohormonal axis, SGLT2 inhibitors may act through more conserved, systemic mechanisms underlying HF [108]. This may explain their beneficial effect across a broader range of EFs, including in patients with HFmrEF and HFpEF [109]. However, caution should be exercised when interpreting the results of clinical trials. The magnitude of the therapeutic effect is not a simple reflection of the biological relevance of a given pathway. It also depends on study design, inclusion criteria, population characteristics, background treatment, endpoint definitions, and follow-up time. Nevertheless, differences in treatment response between HFrEF and HFmrEF may be partially explained by differences in the network architecture of these phenotypes [110,111,112]. In HFrEF, the greater dominance of neurohormonal pathways favors reproducible effects of targeted therapies, whereas in HFmrEF, biological heterogeneity may dilute the therapeutic signal at the population level [113]. This approach has significant implications for clinical practice and study design. Rather than interpreting HFmrEF as a uniform intermediate category between HFrEF and HFpEF, efforts should focus on identifying the dominant disease mechanisms in individual patients. Treatment should be selected not only on the basis of the current LVEF value but also taking into account the EF trajectory, structural cardiac imaging, comorbidities, biomarkers, and features suggesting the activity of specific pathophysiological pathways [114,115,116].

### Biomarkers as Surrogates of Network Activity

If heart failure is viewed as a network disorder, biomarkers may be interpreted not merely as indicators of disease severity but as partial readouts of dominant biological processes. In this framework, circulating biomarkers provide indirect information regarding the relative activity of specific network domains.

Natriuretic peptides predominantly reflect hemodynamic stress and neurohormonal activation. Soluble ST2 and galectin-3 provide information regarding fibrosis and extracellular matrix remodeling. Growth differentiation factor-15 may reflect mitochondrial stress and systemic metabolic dysregulation. High-sensitivity troponins may indicate ongoing myocardial injury, whereas inflammatory mediators such as C-reactive protein and interleukin-6 may represent activation of inflammatory network components.

No single biomarker captures the complexity of heart failure biology.

A major challenge in translating a network-based view of heart failure into clinical practice is the absence of direct tools capable of measuring the relative activity of individual biological pathways in routine patient care. Although the concept of network coherence provides a useful framework for understanding disease organization, clinicians ultimately require practical indicators that can approximate underlying biological states. In this context, circulating biomarkers may serve as accessible surrogates of network activity. Importantly, biomarkers should not be interpreted as isolated disease markers but rather as partial reflections of dominant pathophysiological domains, including neurohormonal activation, fibrosis, inflammation, metabolic stress, endothelial dysfunction, and ongoing myocardial injury. Because no single biomarker captures the complexity of heart failure biology, integrating multiple biomarkers may provide a more comprehensive representation of disease architecture and help identify the pathways that occupy central positions within a given patient’s network. Such an approach may facilitate phenotype refinement, improve risk stratification, and ultimately support mechanism-guided therapeutic decision-making. The major biomarker classes and their potential relationship to dominant network domains are summarized in Table 2.

Importantly, patients rarely exhibit activation of a single domain. Clinical phenotypes are more accurately viewed as dynamic combinations of partially overlapping network states, with therapeutic response depending on the relative dominance of individual biological processes.

However, combinations of biomarkers may provide multidimensional information regarding pathway dominance and biological phenotype. Future studies may therefore focus on integrated biomarker panels that approximate disease-network architecture and improve phenotypic classification beyond EF alone.

It is important to emphasize that no currently available biomarker specifically reflects the activity of a single biological pathway or directly quantifies network organization in heart failure. Natriuretic peptides, cardiac troponins, inflammatory biomarkers, and markers of extracellular matrix turnover are influenced by a variety of physiological and pathological factors, including age, renal dysfunction, obesity, atrial fibrillation, systemic inflammatory diseases, and other comorbidities that are frequently present in patients with heart failure. Consequently, biomarker profiles should be interpreted as indirect indicators of ongoing biological processes rather than definitive measures of network coherence or pathway dominance. The concept of biomarker-guided network phenotyping proposed in this review remains hypothetical and requires prospective validation using integrated molecular, imaging, and clinical data.

## 6. Pharmacological Modulation of Network Architecture

**Figure 7:**
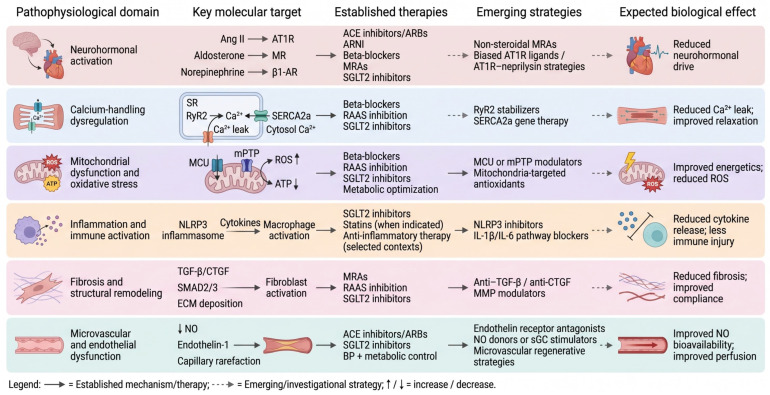
Therapeutic targeting of key pathways in heart failure. Schematic overview of major pathophysiological domains in heart failure, their key molecular targets, established therapies, emerging strategies, and expected biological effects. Solid arrows indicate established mechanisms or therapies, whereas dashed arrows indicate emerging or investigational strategies. ACE—angiotensin-converting enzyme; Ang II—angiotensin II; ARB—angiotensin receptor blocker; ARNI—angiotensin receptor–neprilysin inhibitor; AT1R—angiotensin II type 1 receptor; BP—blood pressure; Ca^2+^—calcium ion; CTGF—connective tissue growth factor; ECM—extracellular matrix; IL—interleukin; MCU—mitochondrial calcium uniporter; MMP—matrix metalloproteinase; MRA—mineralocorticoid receptor antagonist; mPTP—mitochondrial permeability transition pore; NO—nitric oxide; NLRP3—NOD-like receptor family pyrin domain containing 3; RAAS—renin–angiotensin–aldosterone system; ROS—reactive oxygen species; RyR2—ryanodine receptor 2; SERCA2a—sarcoplasmic/endoplasmic reticulum Ca^2+^-ATPase 2a; SGLT2—sodium–glucose cotransporter 2; sGC—soluble guanylate cyclase; SMAD2/3—SMAD family member 2/3; SR—sarcoplasmic reticulum; TGF-β—transforming growth factor beta. Created in BioRender. Mertowski, S. (2026) https://BioRender.com/tc2ats7.

Pharmacological treatment of HF can be interpreted as targeted modulation of key nodes within a complex pathophysiological network. Individual drug classes do not act solely on isolated pathways but influence interconnected processes, including neurohormonal activation, sodium and water metabolism, vascular tone, fibrosis, oxidative stress, cellular metabolism, and left ventricular remodeling. The clinical significance of a given therapy therefore depends on whether its primary mechanism of action aligns with processes central to the disease network of that phenotype. In HFrEF, primary pharmacological treatment is based on modulating pathways that, in many patients, serve as overarching regulators of disease progression. Blocking the renin–angiotensin–aldosterone system, limiting chronic sympathetic activation, inhibiting aldosterone action, and influencing remodeling and volume overload disrupt the mutually reinforcing mechanisms underlying maladaptive remodeling. Therefore, therapies targeting these processes demonstrate more consistent and reproducible clinical benefits in HFrEF [103,124,125,126,127,128] (Figure 7).

Angiotensin-converting enzyme inhibitors (ACEi), angiotensin II receptor blockers (ARBs), and angiotensin receptor–neprilysin inhibitors (ARNIs) target one of the main pathophysiological axes of HFrEF. Reducing angiotensin II-dependent signaling reduces vasoconstriction, sodium retention, fibroblast activation, oxidative stress, and left ventricular remodeling. ARNIs additionally enhance beneficial natriuretic peptide-dependent signaling, which may influence vascular tone, diuresis, natriuresis, and fibrosis. In HFmrEF, the impact of these therapies is likely greatest in patients with an HFrEF-like phenotype, particularly those with preexisting low LVEF, left ventricular dilatation, ischemic cardiomyopathy, or evidence of active remodeling. β-blockers reduce chronic catecholamine toxicity, heart rate, myocardial oxygen demand, arrhythmogenesis, and maladaptive activation of β-adrenergic receptors. Their importance is particularly evident in HFrEF, where chronic sympathetic activation is a dominant component of the disease network. In HFrEF, the benefit of β-blockers may be more dependent on the clinical context, particularly sinus rhythm, preexisting systolic dysfunction, the presence of ischemia, tachycardia, or features of progression towards HFrEF [129].

Mineralocorticoid receptor antagonists (MRAs) limit the effects of chronic aldosterone activation, including sodium retention, fibrosis, vascular inflammation, oxidative stress, and myocardial electrical remodeling. From a network perspective, MRAs operate at the interface among neurohormonal activation, extracellular matrix remodeling, and electrical instability. In HFmrEF, their potential importance may be greater in patients with a remodeling, fibrosis, volume overload or HFrEF-like phenotype, but efficacy and safety require consideration of renal function and the risk of hyperkalemia [130,131,132].

SGLT2 inhibitors occupy a special place in the treatment of HF because their action spans multiple biological domains. These drugs affect sodium metabolism, intravascular volume regulation, renal function, energy metabolism, oxidative stress, inflammation, and likely cardiomyocyte ionic overload [108,133].

Unlike therapies primarily targeting the classic neurohormonal axis, SGLT2 inhibitors may modulate more preserved, systemic mechanisms of heart failure. This may explain their beneficial effects across a broader spectrum of LVEF, including in patients with HFmrEF and HFpEF [97,134,135].

Loop diuretics remain the mainstay of symptomatic treatment for congestion, reducing filling pressures, peripheral edema, and symptoms of fluid overload. However, they are not classically considered disease-modifying treatments in the sense of reducing mortality or reversing remodeling [136].

In the network model, they can be considered an intervention that reduces the hemodynamic consequences of the disease but does not necessarily affect the overarching mechanisms driving progression. However, their clinical importance remains high, especially in patients with congestion symptoms, regardless of EF category.

Select therapies, such as hydralazine with isosorbide dinitrate or ivabradine, have more specific applications. The former affects hemodynamic burden and nitric oxide bioavailability, while the latter reduces sinus rhythm by inhibiting the If current. Their place in treatment depends on specific clinical features, tolerability of basic therapies, and the presence of an appropriate pathophysiological substrate [137,138,139]. In HFmrEF, they should not be considered routine therapies but rather treatments reserved for selected clinical situations. A summary of the major drug classes, their mechanistic targets, and their role in HFrEF and HFmrEF is presented in Table 3.

From the perspective of the proposed model, differences in the strength of recommendations for individual therapies should not be interpreted solely as a simple hierarchy of drug efficacy. They also reflect the extent to which a given mechanism of action aligns with the dominant biological processes in a given patient population. In HFrEF, multiple therapies target central nodes of a relatively coherent disease network, which promotes reproducible clinical outcomes. In HFmrEF, the response depends more on the disease’s biological subtype; therefore, treatment should be interpreted in the context of the EF trajectory, left ventricular structure, comorbidities, biomarkers, and the likely dominance of specific pathways.

## 7. Device and Interventional Therapy as Modulation of the Structural Substrate

Device and interventional therapy for HF differ from pharmacotherapy in that their primary goal is not the direct modulation of systemic signaling pathways, but rather the correction of a specific structural, electrophysiological, or hemodynamic substrate. Such substrates include, among others, myocardial scarring and fibrosis, ventricular dyssynchrony, conduction abnormalities, arrhythmogenic remodeling, ischemia, myocardial hibernation, and significant valvular defects. The effectiveness of these interventions therefore depends on whether a given substrate actually plays a significant role in the disease network of a given patient.

In HFrEF, chronic neurohormonal activation, left ventricular remodeling, fibrosis, and disorders of calcium metabolism promote the development of an arrhythmogenic substrate and unfavorable electrical remodeling. In selected patients, an implantable cardioverter–defibrillator (ICD) reduces the risk of sudden cardiac death by interrupting dangerous ventricular arrhythmias. From a network perspective, an ICD does not directly modify the mechanisms underlying remodeling but limits one of the most serious consequences of structural and electrophysiological remodeling of the myocardium.

Cardiac resynchronization therapy (CRT) targets another element of the disease network: electromechanical dyssynchrony. In patients with significant conduction disturbances, particularly left bundle branch block and a widened QRS complex, dyssynchrony exacerbates left ventricular mechanical failure, increases mitral regurgitation, and enhances remodeling. CRT can improve contractile coordination, increase hemodynamic efficiency, and promote reverse remodeling. However, its effectiveness is strictly dependent on the presence of an appropriate electrophysiological substrate and therefore cannot be interpreted solely through the lens of left ventricular ejection fraction.

In HFmrEF, the role of device therapy is more selective. An LVEF of 41–49% alone does not provide a sufficient basis for routine use of an ICD or CRT. However, some patients with HFmrEF, especially after improvement from previous HFrEF or with progressive systolic dysfunction, may retain a substrate resembling HFrEF: ischemic scar, fibrosis, left ventricular dilatation, conduction disturbances, or a significant risk of arrhythmia. In such cases, the decision to administer device therapy should be based on a comprehensive risk assessment, history of EF, structural cardiac findings, electrocardiographic findings, and current indications, rather than on the diagnosis of HFmrEF alone.

Coronary and valvular interventions further demonstrate the limitations of classification based solely on EF. In patients with ischemic cardiomyopathy, revascularization can modify the course of the disease by improving perfusion, limiting ischemia, or restoring function to the hibernating myocardium. Similarly, correction of significant valvular disease can reduce volume or pressure overload, interrupt maladaptive remodeling, and improve symptoms. In both cases, the key is identifying the modifiable mechanism driving heart failure, not the LVEF category itself. A summary of the main equipment and structural interventions, along with their interpretation in the network model, is presented in Table 4.

## 8. Guideline Interpretation and Phenotype-Targeted Clinical Strategy

Clinical guidelines for HF are typically presented as treatment algorithms based on ejection fraction categories and the strength of evidence from clinical trials. However, from a network biology perspective, they can also be interpreted as an indirect reflection of the degree to which a given pathophysiological mechanism is reproducible and therapeutically relevant in a given patient population. Strong recommendations are based not only on a drug’s formal efficacy but also on the targeted biological axis that occupies a central place in the disease architecture in a large portion of the studied population [140].

In HFrEF, the consistency of treatment recommendations reflects both the extensive evidence base and the relatively high mechanistic consistency of this phenotype. Numerous disease-modifying therapies target dominant pathways, including the RAAS, the sympathetic nervous system, aldosterone, left ventricular remodeling, sodium retention, and volume overload [113,114,124,141,142,143]. Because these processes are strongly linked to HFrEF progression, modulating them yields reproducible clinical benefits, including reduced risk of hospitalization, improved prognosis, and slower disease progression.

In HFmrEF, the situation is more complex. Weaker or more conditional recommendations for some HFrEF-specific therapies should not be interpreted as evidence of their lack of efficacy in all patients with HFmrEF. Rather, they reflect the biological heterogeneity of this category and the fact that classic neurohormonal pathways do not occupy a central place in the disease network in every patient [78]. Treatment directed at the RAAS, SNS, or aldosterone may be particularly justified in patients with preexisting low LVEF, left ventricular dilatation, ischemic cardiomyopathy, high natriuretic peptide levels, or evidence of progression towards HFrEF.

SGLT2 inhibitors occupy a different position. Their benefits observed across a wide range of ejection fractions can be attributed to their effects on more conserved, systemic axes of HF biology, including renal function, volume regulation, metabolism, oxidative stress, and inflammation [108,109,144]. Therefore, SGLT2 inhibitors are a particularly important component of HFmrEF treatment, regardless of whether the patient’s phenotype is closer to HFrEF or HFpEF, although their clinical effect may still be modulated by comorbidities and risk profile.

A phenotypically targeted clinical strategy in HFmrEF should begin with defining the disease trajectory. The key question is not simply whether the current LVEF is within the 41–49% range, but rather where this value comes from. The clinical significance of a patient with a previous LVEF ≤ 40% who improved after treatment differs from that of a patient with a progressive decline in EF, and yet differs from that of a patient with hypertension, obesity, diabetes, chronic kidney disease, atrial fibrillation, and predominant diastolic dysfunction. In each of these cases, the same EF value may reflect a different biological architecture.

In clinical practice, the assessment of HFmrEF should be based on several complementary layers of information: LVEF history, left ventricular structural image, the presence of scarring or fibrosis, biomarker profile, cardiac rhythm, comorbidities, congestion severity, and response to prior treatment. Patients with HFmrEF who have improved from prior HFrEF should typically continue disease-modifying therapy, as improved EF does not necessarily indicate resolution of the underlying pathophysiological processes. Patients with signs of systolic progression require early intensification of therapy typical of HFrEF and monitoring of remodeling. However, patients with HFmrEF with an HFpEF-like profile require special emphasis on managing comorbidities, controlling blood pressure, reducing congestion, optimizing metabolism, treating atrial fibrillation, and using therapies effective across a broad spectrum of EF, particularly SGLT2 inhibitors.

This approach does not replace guidelines but facilitates their more precise application. While EF categories remain useful as a starting point, therapeutic decisions should be interpreted in the context of the dominant biological phenotype. In HFrEF, rapid implementation and optimization of disease-modifying treatments are warranted, as targeted pathways often occupy central positions in the pathological network. In HFmrEF, however, it is essential to combine EF-based recommendations with an assessment of the mechanisms driving the disease in a given patient. From a future research perspective, this approach highlights the need to design clinical trials that do not rely solely on rigid EF thresholds but instead account for LVEF trajectory, structural phenotype, biomarkers, imaging, and comorbidities. This would allow better identification of patient subgroups in which a given pathway is truly dominant, thereby increasing the likelihood of detecting a true therapeutic effect.

## 9. An Integrative Model of HF as a Multilevel Disorder

HF can be understood as a multilevel disorder in which molecular, cellular, tissue, organ, and systemic processes form a dynamic network of interdependencies. In this approach, the clinical phenotype is not a simple consequence of a single mechanism, but rather the result of interactions between neurohormonal activation, inflammation, oxidative stress, mitochondrial dysfunction, calcium metabolism disorders, extracellular matrix remodeling, endothelial dysfunction, hemodynamic stress, renal function, metabolism, and comorbidities [145].

Each of these elements may play a different role depending on the etiology of the disease, the stage of heart failure, and the patient’s phenotype. At the systemic level, HF can be initiated and sustained by various factors, such as myocardial ischemia, hypertension, primary cardiomyopathies, valvular heart disease, arrhythmias, obesity, diabetes, chronic kidney disease, aging, and chronic inflammation. These factors activate distinct, yet partially overlapping, biological pathways. Their combined effects can include increased filling pressures, congestion, reduced exercise tolerance, left ventricular remodeling, altered cardiac geometry, and reduced or relative preservation of LVEF. This means that ejection fraction is a clinically useful yet definitive marker of the disease network, rather than a direct definition of the disease mechanism.

At the molecular and cellular levels, HF phenotypes differ less in the presence or absence of specific pathways than in their relative importance and mutual organization. Activation of the RAAS and SNS can coexist with calcium metabolism disorders, mitochondrial dysfunction, oxidative stress, and fibrosis. Inflammation can exacerbate endothelial dysfunction, limit nitric oxide bioavailability, increase myocardial stiffness, and promote fibroblast activation. Metabolic disorders can exacerbate mitochondrial dysfunction, lipotoxicity, and oxidative stress, while renal dysfunction can exacerbate fluid overload, neurohormonal activation, and inflammation. This creates a feedback network in which primary and secondary processes mutually reinforce each other.

In HFrEF, the pathophysiological network often adopts a more coherent and hierarchical organization. Activation of the RAAS and SNS, left ventricular remodeling, fibrosis, calcium metabolism disorders, and mitochondrial dysfunction constitute a system of mutually reinforcing processes. In this system, neurohormonal pathways function as master regulators whose modulation leads to effects beyond the drug’s direct target. Blocking the RAAS, limiting sympathetic activation, or inhibiting aldosterone can indirectly influence remodeling, oxidative stress, hemodynamic stress, electrical stability, and cellular function. This may explain the reproducibility of therapeutic benefits observed in HFrEF.

HFmrEF occupies a more complex position within this model. It is not simply an “intermediate” range of EF, but a category encompassing diverse biological trajectories. In some patients, it represents a state after improvement from previous HFrEF, in which residual elements of the former disease network persist, such as fibrosis, altered β-adrenergic sensitivity, incomplete calcium normalization, or persistent neurohormonal activation. In others, it may represent a stage of progression towards HFrEF, before full consolidation of neurohormonal, metabolic, and structural pathways occurs. In another group, HFmrEF may resemble HFpEF, with a predominant role of chronic inflammation, endothelial dysfunction, microcirculatory dysfunction, metabolic disease, chronic kidney disease, and atrial fibrillation. Therefore, in patients with HFmrEF, a similar LVEF value may mask a different biological organization of the disease.

A key element of the integrative model is the concept of pathway dominance. The same biological process may have different significance depending on the patient’s phenotype. Activation of the RAAS may be a central mechanism of progression in patients with HFrEF or HFmrEF with an HFrEF-like profile but may be less important in patients with HFmrEF dominated by obesity, microcirculatory dysfunction, inflammation, and atrial fibrillation. Similarly, inflammation may be a secondary process in classic HFrEF, but one of the main mechanisms driving the disease in HFpEF-like phenotypes.

The therapeutic response therefore depends not only on the presence of a given pathway but also on its position in the disease network.

The proposed integrative model combines three levels of interpretation. First, it indicates that LVEF should be treated as an outcome parameter and interpreted in the context of the patient’s medical history, the dynamics of EF changes, cardiac imaging, biomarkers, heart rhythm, and comorbidities. Second, it emphasizes the importance of pathway dominance, meaning which biological processes actually control the course of the disease in a given patient. Third, it allows for understanding therapeutic response as a consequence of matching the treatment’s mechanism of action to the central elements of the disease network. In this approach, the same medication can have different clinical implications depending on whether its target mechanism is dominant, secondary, or merely concomitant.

In clinical practice, this approach requires integrating several layers of information. The first is the LVEF trajectory: the current EF value has a different meaning in a patient with previous HFrEF and improved systolic function, in a patient with progressive EF decline, and in a patient with persistently mildly reduced EF and prevalent comorbidities. The second layer is the structural cardiac phenotype, encompassing left ventricular size, hypertrophy, geometry, right ventricular function, left atrial size, and the presence of scar tissue, fibrosis, or strain abnormalities. The third layer comprises biomarkers reflecting neurohormonal activation, myocardial damage, inflammation, fibrosis, renal function, and metabolic status. The fourth layer comprises comorbidities such as hypertension, diabetes, obesity, chronic kidney disease, atrial fibrillation, coronary artery disease, and valvular disease. Biomarkers may play a significant role in the transition from EF-based classification to a mechanistic classification. Natriuretic peptides reflect hemodynamic overload and wall stress; troponins may indicate myocardial damage; inflammatory markers indicate immune activation; and renal and metabolic parameters indicate involvement of the cardiorenal–metabolic axis. However, no single biomarker defines the entire disease network. Their value increases only when combined with imaging, clinical characteristics, and disease dynamics.

Cardiac imaging constitutes the second key layer of phenotyping. Echocardiography allows for the assessment of LVEF, cardiac chamber dimensions, hypertrophy, diastolic function, right ventricular function, pulmonary pressures, and valvular disease. Analysis of myocardial strain can reveal subclinical dysfunction not evident in EF alone. Cardiac magnetic resonance imaging allows for the assessment of scar tissue, fibrosis, inflammation, extracellular volume, and tissue characteristics. Incorporating this data allows better differentiation between patients with predominant systolic remodeling and those in whom stiffness, microcirculation, ischemia, or comorbidities play a major role.

An integrative model also has implications for the interpretation of clinical trials. A randomized trial can be viewed as a population-based trial aimed at modifying a specific pathway. If the population is relatively biologically homogeneous and the drug targets a dominant mechanism, the therapeutic effect should be easier to detect. However, if the study includes a heterogeneous population in which the same EF range results from different biological configurations, the effect may be attenuated or limited to specific subgroups. This is particularly true for HFmrEF, where a single EF category encompasses patients with different disease trajectories and mechanisms.

From this perspective, differences in study results and the strength of treatment recommendations do not necessarily imply simple differences in drug efficacy. Rather, they may reflect the extent to which the treatment mechanism aligns with the biology of the study population. Therapies targeting the RAAS, SNS, or aldosterone show the greatest coherence when these pathways are central to the disease network. SGLT2 inhibitors, however, may have a broader effect, as they affect more systemic axes, including volume management, renal function, metabolism, and inflammation.

**Figure 8:**
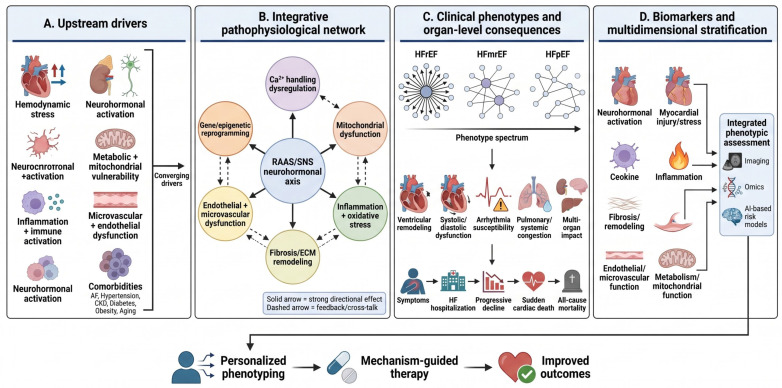
Integrative multilevel model of HF progression and phenotypic heterogeneity. Schematic representation of HF as a multilevel disorder linking upstream drivers, an integrative pathophysiological network, clinical phenotypes, organ-level consequences, biomarkers, and multidimensional stratification. The model illustrates how converging biological and clinical inputs may support personalized phenotyping, mechanism-guided therapy, and improved outcomes. AF—atrial fibrillation; CKD—chronic kidney disease; ECM—extracellular matrix; HF—heart failure; HFmrEF—HF with mildly reduced ejection fraction; HFpEF—HF with preserved ejection fraction; HFrEF—HF with reduced ejection fraction; LV—left ventricular; RAAS—renin–angiotensin–aldosterone system; SNS—sympathetic nervous system. Arrow notation: Solid black arrows indicate the principal direction of biological and clinical progression, linking upstream drivers to the integrated pathophysiological network, clinical phenotypes, organ-level consequences, biomarkers, and personalized therapeutic strategies. Converging arrows indicate the integration of multiple biological pathways, clinical characteristics, imaging findings, and biomarker information into multidimensional patient phenotyping. Diverging arrows indicate biological heterogeneity, illustrating that common upstream mechanisms may give rise to distinct heart failure phenotypes and different clinical trajectories. Bidirectional arrows (↔) indicate reciprocal interactions and dynamic feedback between biological mechanisms, clinical phenotype, and disease progression. Dashed black arrows indicate indirect associations, modulatory effects, or complementary interactions that influence, but do not solely determine, disease phenotype or therapeutic response. Upward arrows (↑) indicate increased activation, predominance, or amplification of biological processes. Downward arrows (↓) indicate reduced activity, attenuation, or suppression of biological processes. Created in BioRender. Mertowski, S. (2026) https://BioRender.com/tc2ats7.

This interpretation allows for the integration of mechanistic data, research findings, and clinical practice into a single conceptual framework (Figure 8).

However, it should be emphasized that the proposed model remains a conceptual construct, not a definitive predictive system. It does not assume that every patient with HFrEF has identical biology or that every patient with HFmrEF has low network coherence. HFrEF also encompasses diverse etiologies and subtypes, and some patients with HFmrEF may retain distinct HFrEF biology. Rather, the model indicates that at the population level, HFrEF more often exhibits a predominance of neurohormonal remodeling pathways, whereas HFmrEF more often exhibits mixed, transient, and comorbidity-dependent phenotypes. From a practical perspective, this model supports the transition from categorical classification to a mechanistically driven approach. This does not mean abandoning LVEF, as it remains a fundamental and useful clinical parameter. However, it does require interpreting it within a broader biological context. In the future, integrating EF, biomarkers, imaging, omics data, and computational methods may enable more accurate HF subtyping and more precise treatment selection. Ultimately, an integrative model of HF as a multilevel disorder allows for the integration of pathophysiology, clinical phenotype, and therapeutic response. Its primary value lies in shifting the focus from EF alone to the question of what biological processes lead to a given phenotype and which of these can be effectively modified. This approach provides a foundation for the further development of mechanistic classification and precision cardiology.

## 10. Future Research Directions

The limitations of HF classification based primarily on ejection fraction indicate the need to develop more multidimensional, mechanistically focused patient stratification models. LVEF remains a clinically useful parameter, but it does not provide a clear definition of the dominant biological processes underlying disease progression and therapeutic response. Future research should therefore focus not only on further refining EF thresholds but primarily on identifying the mechanisms that occupy a central place in the disease network in individual patients.

One of the most important directions is the development of phenotyping strategies that integrate clinical, imaging, biomarker, and molecular data. Combining information on LVEF trajectory, left ventricular dimensions and geometry, the presence of scar tissue or fibrosis, diastolic function, strain, natriuretic peptide profile, markers of myocardial damage, inflammation, renal dysfunction, and metabolic disorders may be particularly valuable [146,147]. This approach could enable the differentiation of patients with HFmrEF after improvement from those with progression to systolic dysfunction and from those with an HFpEF-like phenotype.

Omics technologies, including transcriptomics, proteomics, metabolomics, and epigenomics, may play a significant role in future HF classification. These data may reveal molecular differences not visible in traditional EF-based classification, especially in heterogeneous categories such as HFmrEF [81,148,149,150,151].

Integrating omics findings with imaging and biomarkers may enable the identification of reproducible biological modules, such as dominance of neurohormonal signaling, fibrosis, inflammation, mitochondrial dysfunction, calcium metabolism disorders, or the cardiorenal–metabolic axis.

Another area requiring development is research on the quantitative assessment of network coherence and pathway dominance. In this paper, these concepts are conceptual, but in the future, they could be translated into measurable indicators based on multivariate data. Computational models, network analysis, and machine learning methods can help identify groups of patients with similar biological architecture and predict response to specific therapies. However, their clinical utility will require prospective validation, standardization of input data, and demonstration of advantages over simple EF classification.

Future clinical trials should also more extensively consider mechanistic enrichment of study populations. Traditional inclusion criteria based on EF ranges can lead to grouping patients with different biological configurations, weakening the therapeutic signal. In HFmrEF, it would be particularly important to design studies or subgroup analyses that account for prior LVEF, the direction of left ventricular EF change, the presence of left ventricular remodeling, ischemia, fibrosis, heart rhythm, metabolic diseases, renal function, and biomarkers. This approach could more effectively identify which patients are best served by HFrEF-specific therapies and which are best served by strategies targeting systemic processes.

In parallel, further research is needed into therapeutic targets beyond classical neurohormonal modulation. Despite significant progress in the treatment of HFrEF, residual risk remains high, suggesting the involvement of additional mechanisms, such as mitochondrial dysfunction, metabolic disturbances, inflammation, NLRP3 inflammasome activation, fibrosis, and endothelial dysfunction [152,153,154].

Translating mechanistic models into clinical practice also remains a significant challenge. Even the most advanced multidimensional approaches will only be useful if they prove applicable to routine care, cost-effective, and interpretable for clinicians. Therefore, future research should encompass not only the development of complex predictive models but also their simplification into practical decision-making algorithms that could support treatment selection in patients with HFrEF and HFmrEF. Ultimately, further development of HF classification should aim to develop models that combine the utility of simple clinical parameters, such as LVEF, with deeper biological characterization of the disease. Particularly in HFmrEF, where a single EF category encompasses diverse trajectories and mechanisms, integrating clinical, imaging, biomarker, and molecular data can provide the basis for more precise patient stratification and the design of therapies tailored to the prevailing pathophysiological processes.

Importantly, the proposed framework should not be interpreted as inferring pathway dominance from the observed success or failure of individual therapies. Instead, it generates the hypothesis that independently measurable features of biological network organization determine therapeutic responsiveness. Clinical trial outcomes are therefore regarded as observations that are compatible with the proposed model rather than evidence establishing its validity. The ultimate evaluation of this hypothesis will require prospective studies in which patient-specific network architecture is characterized before treatment initiation and subsequently correlated with therapeutic response.

### 10.1. Future Directions: Multi-Omics and Network Phenotyping

The increasing availability of high-throughput molecular technologies offers an opportunity to move beyond phenotype classification based solely on ventricular function. Transcriptomics, proteomics, metabolomics, epigenomics, and single-cell sequencing now permit detailed characterization of disease-associated biological networks across multiple organizational levels.

Future heart failure classification systems may integrate molecular signatures with imaging findings, circulating biomarkers, disease trajectory, and clinical phenotype. Rather than assigning patients to a single EF-defined category, such approaches may identify biologically coherent endotypes characterized by dominant neurohormonal, inflammatory, fibrotic, metabolic, or endothelial mechanisms.

Artificial intelligence and machine learning methods may further facilitate integration of multidimensional datasets and enable reconstruction of patient-specific network architecture. Such approaches could improve risk stratification, identify therapeutic targets, and support precision medicine strategies.

Importantly, HFmrEF may represent one of the most informative populations for developing these approaches because it occupies a biological transition zone in which multiple disease trajectories intersect. Understanding this heterogeneity may provide insights extending beyond HFmrEF itself and contribute to a broader redefinition of heart failure classification.

Although the present framework remains conceptual, contemporary systems biology provides a plausible pathway for its prospective validation. Rather than inferring network organization retrospectively from therapeutic outcomes, future studies could reconstruct patient-specific biological interaction networks using multimodal molecular datasets, including transcriptomic, proteomic, metabolomic, and imaging-derived information. Computational network reconstruction methods—such as weighted gene co-expression network analysis (WGCNA), Bayesian network inference, protein–protein interaction mapping, graph-based machine learning, or causal network modeling—could then be used to identify regulatory hubs and characterize the overall architecture of disease networks.

Within these reconstructed networks, quantitative descriptors such as node centrality, modularity, clustering coefficient, network entropy, and pathway enrichment scores could serve as candidate measures of network organization. These computational metrics may ultimately allow operational assessment of the concepts proposed in this review, including network coherence and pathway dominance. Such biological descriptors could then be correlated prospectively with clinical phenotypes, disease trajectories, and therapeutic responses to determine whether patients exhibiting highly centralized neurohormonal networks derive greater benefit from upstream neurohormonal inhibition than patients whose disease biology is distributed across inflammatory, metabolic, and microvascular pathways. Accordingly, the proposed framework should be regarded not as a retrospective explanation of existing clinical trial results but as a hypothesis-generating research program that provides experimentally testable predictions.

### 10.2. Limitations of the Proposed Framework

Several limitations should be acknowledged. First, the concept of network coherence proposed in this review represents a conceptual and hypothesis-generating construct rather than a validated quantitative parameter. At present, no accepted methodology exists for measuring network coherence in individual patients.

Second, biological pathways interact dynamically over time and cannot be fully separated into discrete categories. Considerable overlap exists among inflammatory, neurohormonal, metabolic, and fibrotic processes, limiting the precision of any simplified classification framework.

Third, the proposed model is derived from integration of mechanistic and clinical evidence rather than direct network reconstruction studies. Consequently, prospective validation using systems biology approaches, multi-omics datasets, and longitudinal patient cohorts is required.

Fourth, therapeutic response is influenced not only by pathway dominance but also by age, comorbidity burden, genetic background, treatment adherence, and environmental factors. The framework should therefore be viewed as complementary to current clinical classification systems rather than a replacement for them.

Finally, an important limitation of the proposed framework is that current biomarker assessment does not allow direct characterization of the biological architecture underlying individual heart failure phenotypes. Future advances in multi-omics technologies, molecular imaging, and systems biology may enable more precise characterization of disease networks and facilitate.

## 11. Conclusions

This review proposes HF classification based solely on LVEF does not fully capture the disease’s biological complexity. LVEF remains an important and practical clinical parameter, but it should be interpreted as the end result of multiple interconnected processes, rather than as a direct definition of the pathophysiological mechanism. Similar EF values may result from distinct molecular, cellular, structural, and systemic configurations, which have significant implications for prognosis and therapeutic response.

This review proposes a conceptual network-based framework for interpreting heart failure with reduced and mildly reduced ejection fraction beyond conventional ejection fraction-based classification. Rather than viewing HFrEF and HFmrEF as fixed categories defined solely by ventricular systolic function, the proposed framework emphasizes differences in the organization and interaction of biological pathways that may contribute to phenotypic heterogeneity and variable therapeutic response.

The framework is intended as a hypothesis-generating model rather than a validated biological classification. Its primary purpose is to integrate current mechanistic and clinical evidence into a coherent conceptual perspective that may facilitate interpretation of existing data, support the development of biologically informed therapeutic strategies, and stimulate future translational and clinical research aimed at validating mechanism-based approaches to heart failure phenotyping.

## Figures and Tables

**Table 1:** Major biological phenotypes of HFmrEF and their clinical–therapeutic relevance.

HFmrEF Phenotype	Typical Clinical Trajectory	Dominant Biological Mechanisms	Features Suggesting the Phenotype	Therapeutic Implications
HFmrEF after recovery from previous HFrEF/HFimpEF	Improvement in LVEF after previous systolic dysfunction, usually following treatment or removal of the causative factor	Persistent neurohormonal activation, residual fibrosis, extracellular matrix remodeling, altered β-adrenergic sensitivity, incomplete normalization of calcium handling	Documented previous LVEF ≤ 40%, improvement of EF to the 41–49% range, history of dilated or ischemic cardiomyopathy, LV dilation, elevated natriuretic peptides, good response to guideline-directed medical therapy	Continue full disease-modifying therapy; avoid withdrawal of treatment despite EF improvement; monitor for recurrence of systolic dysfunction
HFmrEF as early or progressive systolic dysfunction	Gradual deterioration of LV function or transition from borderline EF values toward HFrEF	Increasing RAAS/SNS activation, early LV remodeling, oxidative stress, initial SERCA2a/RyR2 abnormalities, mitochondrial dysfunction	Decline in LVEF on serial assessments, LV dilation, reduced strain, high BNP/NT-proBNP, ischemic cardiomyopathy, scar on CMR, tachycardia or volume overload	Early intensification of HFrEF-type therapy; control of ischemia, rhythm, and blood pressure; close monitoring of EF progression and remodeling
HFmrEF with an HFpEF-like phenotype	Persistent mildly reduced LVEF with predominant congestion, diastolic dysfunction, and comorbidity-driven disease features	Chronic low-grade inflammation, endothelial dysfunction, microvascular impairment, reduced nitric oxide bioavailability, impaired cGMP–PKG signaling, obesity, insulin resistance, chronic kidney disease, atrial fibrillation	Hypertension, obesity, diabetes, chronic kidney disease, atrial fibrillation, LV hypertrophy, left atrial enlargement, features of diastolic dysfunction, absence of marked LV dilation, signs of congestion	Treat comorbidities; control blood pressure and heart rhythm; reduce congestion; promote weight reduction and metabolic optimization; consider SGLT2 inhibitors as broad systemic therapy

Abbreviations: BNP—B-type natriuretic peptide; **β**—beta (β-adrenergic)**;** cGMP–PKG—cyclic guanosine monophosphate–protein kinase G; CMR—cardiac magnetic resonance; EF—ejection fraction; HFimpEF—heart failure with improved ejection fraction; HFmrEF—heart failure with mildly reduced ejection fraction; HFpEF—heart failure with preserved ejection fraction; HFrEF—heart failure with reduced ejection fraction; LV—left ventricular; LVEF—left ventricular ejection fraction; NT-proBNP—N-terminal pro-B-type natriuretic peptide; RAAS—renin–angiotensin–aldosterone system; RyR2—ryanodine receptor 2; SERCA2a—sarcoplasmic/endoplasmic reticulum Ca^2+^-ATPase 2a; SGLT2—sodium–glucose cotransporter 2; SNS—sympathetic nervous system.

**Table 2:** Biomarker-informed interpretation of dominant network domains in heart failure [117,118,119,120,121,122,123].

Dominant Network Domain	Principal Biological Processes	Representative Biomarkers *	Expected Phenotypic Features	Potential Therapeutic Implications
Neurohormonal activation	RAAS and sympathetic nervous system activation, sodium retention, hemodynamic stress, ventricular remodeling	BNP, NT-proBNP, plasma renin activity, aldosterone, norepinephrine	HFrEF-like phenotype, ventricular dilation, elevated filling pressures, progressive remodeling	Optimization of ACEi/ARB/ARNI, β-blockers, MRAs, decongestive strategies
Fibrotic/remodeling domain	Extracellular matrix turnover, fibroblast activation, collagen deposition, ventricular stiffening	sST2, galectin-3, procollagen peptides (PINP, PIIINP), matrix metalloproteinases	Increased ventricular stiffness, adverse remodeling, arrhythmogenic substrate, incomplete reverse remodeling	Anti-remodeling therapies, RAAS inhibition, MRA therapy, intensified follow-up
Inflammatory domain	Cytokine activation, innate immune signaling, inflammasome activity, chronic low-grade inflammation	CRP, IL-6, TNF-α, soluble TNF receptors	Obesity-associated HF, HFpEF-like features, frailty, systemic comorbidity burden	Aggressive management of comorbidities, metabolic optimization, investigation of anti-inflammatory strategies
Metabolic–mitochondrial domain	Impaired oxidative phosphorylation, altered substrate utilization, energetic deficiency, oxidative stress	GDF-15, FGF-21, ketone-related metabolic markers, lactate	Diabetes-associated HF, obesity, exercise intolerance, impaired energetic reserve	SGLT2 inhibitors, metabolic interventions, weight reduction, optimization of insulin resistance
Endothelial–microvascular domain	Endothelial dysfunction, impaired NO bioavailability, reduced cGMP–PKG signaling, microvascular rarefaction	VCAM-1, ICAM-1, endothelin-1, asymmetric dimethylarginine (ADMA)	HFpEF-like phenotype, hypertension, atrial fibrillation, impaired relaxation, exercise intolerance	Blood pressure control, vascular risk reduction, rhythm management, therapies targeting endothelial dysfunction
Myocardial injury domain	Ongoing cardiomyocyte injury, apoptosis, ischemic damage, maladaptive stress responses	High-sensitivity cardiac troponin T/I, heart-type fatty acid-binding protein	Progressive systolic dysfunction, ischemic substrate, higher risk of adverse outcomes	Identification and treatment of ischemia, optimization of disease-modifying therapy, closer surveillance

* Biomarkers are not disease-specific and should be interpreted as components of an integrated biological profile rather than as isolated indicators of pathway activity. Abbreviations: ACEi—angiotensin-converting enzyme inhibitor; ADMA—asymmetric dimethylarginine; ARB—angiotensin II receptor blocker; ARNI—angiotensin receptor–neprilysin inhibitor; BNP—B-type natriuretic peptide; cGMP—cyclic guanosine monophosphate; CRP—C-reactive protein; FGF-21—fibroblast growth factor 21; GDF-15—growth differentiation factor 15; HF—heart failure; HFpEF—heart failure with preserved ejection fraction; HFrEF—heart failure with reduced ejection fraction; ICAM-1—intercellular adhesion molecule 1; IL-6—interleukin 6; MRA—mineralocorticoid receptor antagonist; NO—nitric oxide; NT-proBNP—N-terminal pro-B-type natriuretic peptide; PIIINP—procollagen type III N-terminal propeptide; PINP—procollagen type I N-terminal propeptide; PKG—protein kinase G; RAAS—renin–angiotensin–aldosterone system; SGLT2—sodium–glucose cotransporter 2; sST2—soluble suppression of tumorigenicity 2; TNF-α—tumor necrosis factor alpha; VCAM-1—vascular cell adhesion molecule 1.

**Table 3:** Major drug classes in HF from a mechanistic network-based perspective.

Drug Class	Main Mechanistic Targets	Relevance in HFrEF	Relevance in HFmrEF	Network-Based Interpretation
ACEi/ARB/ARNI	Inhibition of angiotensin II signaling; reduction of vasoconstriction, sodium retention, fibrosis, oxidative stress, and remodeling; ARNI additionally enhance natriuretic peptide signaling	One of the core therapeutic axes in HFrEF; targets the dominant RAAS axis and LV remodeling	Greatest relevance in patients with an HFrEF-like phenotype, previous low EF, LV dilation, or ischemic cardiomyopathy	Modulation of an upstream neurohormonal node; greater effect when RAAS acts as a central regulator of the disease network
β-blockers	Reduction of chronic sympathetic activation, catecholamine toxicity, tachycardia, myocardial oxygen demand, and arrhythmogenesis	Key therapy in HFrEF, particularly in the presence of chronic SNS activation and reduced contractile reserve	Benefit is more likely in sinus rhythm, previous HFrEF, ischemia, tachycardia, or features of progressive systolic dysfunction	Effective when adrenergic signaling is one of the dominant mechanisms sustaining disease progression
MRA	Blockade of aldosterone signaling; reduction of sodium retention, fibrosis, vascular inflammation, oxidative stress, and electrical remodeling	Important component of HFrEF therapy, affecting remodeling, fibrosis, and arrhythmic risk	Potential relevance in patients with a fibrotic-remodeling or HFrEF-like phenotype; limitations include CKD and hyperkalemia	Acts on a node linking neurohormonal activation, fibrosis, and electrical instability
SGLT2 inhibitors	Regulation of volume status, natriuresis, improvement of renal function, and effects on energetic metabolism, oxidative stress, inflammation, and ionic homeostasis	Effective in HFrEF as part of disease-modifying therapy	Particularly relevant in HFmrEF because of benefits across a broad EF spectrum and a multidirectional mechanism of action	Modulation of a systemic cardio–renal–metabolic axis; potentially less dependent on a single EF-defined phenotype
Loop diuretics	Reduction of congestion, filling pressures, volume overload, and edematous symptoms	Fundamental symptomatic treatment for congestion; without clear disease-modifying effects on remodeling	Equally important for symptom control, particularly in patients with AF, CKD, hypertension, obesity, and volume overload	Modulation of the hemodynamic consequences of disease rather than the main mechanisms of progression
Hydralazine–isosorbide dinitrate	Arterial and venous vasodilation, reduction of loading conditions, and increased NO availability	Selected therapy in specific patient groups or when RAAS blockade is not tolerated	Not routinely used; may be relevant in selected clinical indications	Acts on hemodynamic loading and NO signaling; effect depends on the presence of an appropriate substrate
Ivabradine	Reduction of sinus heart rate through inhibition of the If current without negative inotropic effects	Selected therapy in patients with HFrEF, sinus rhythm, and persistent tachycardia despite β-blocker therapy	Limited relevance; possible role in HFrEF-like HFmrEF with persistent sinus tachycardia	Targets a specific hemodynamic–electrophysiological node rather than the broader disease network

**Abbreviations:** ACEi—angiotensin-converting enzyme inhibitors; AF—atrial fibrillation; ARB—angiotensin receptor blockers; ARNI—angiotensin receptor–neprilysin inhibitors; β—beta (β-blockers, beta-adrenergic receptor blockers); CKD—chronic kidney disease; EF—ejection fraction; HFmrEF—HF with mildly reduced ejection fraction; HFrEF—HF with reduced ejection fraction; LV—left ventricular; LVEF—left ventricular ejection fraction; MRA—mineralocorticoid receptor antagonists; NO—nitric oxide; RAAS—renin–angiotensin–aldosterone system; SGLT2—sodium–glucose cotransporter 2; SNS—sympathetic nervous system.

**Table 4:** Device-based and interventional therapies from a mechanistic network-based perspective.

Intervention	Main Substrate/Mechanistic Target	Relevance in HFrEF	Relevance in HFmrEF	Network-Based Interpretation
ICD	Risk of sudden arrhythmic death related to scar, fibrosis, electrical instability, and low EF	Reduces the risk of sudden cardiac death in selected patients; particularly relevant in the presence of a persistent arrhythmogenic substrate	Not routinely indicated solely on the basis of EF 41–49%; should be considered in the presence of previous low EF, substantial scar burden, arrhythmias, or other guideline-based indications	Limits a major consequence of electrical remodeling but does not address the primary mechanisms driving structural remodeling
CRT	Electromechanical dyssynchrony, conduction abnormalities, and inefficient LV contraction	Improves contraction coordination and may promote reverse remodeling in patients with an appropriate QRS profile	Relevant only when a conduction substrate or pacing indication is present; not determined by HFmrEF status alone	Effective when dyssynchrony is an important node contributing to impaired ventricular function
Revascularization	Ischemia, hibernating myocardium, ischemic scar, and myocardial viability	May modify disease trajectory in ischemic cardiomyopathy depending on coronary anatomy, symptoms, and myocardial viability	Important when HFmrEF results from ischemia, myocardial stunning, or hibernating myocardium	Targets a modifiable causal mechanism independently of EF category
Valvular interventions	Volume or pressure overload caused by clinically significant valvular disease	May reduce hemodynamic load and remodeling when valvular disease is an important contributor to HF	Relevance depends on valvular lesion severity, anatomy, symptoms, and Heart Team assessment rather than EF range alone	Remove or reduce a structural driver of the disease network
Arrhythmia treatment/rhythm control	Atrial fibrillation, tachycardia-induced cardiomyopathy, and loss of atrioventricular synchrony	May improve symptoms and ventricular function, particularly when arrhythmia contributes to HF worsening	Particularly relevant in HFmrEF and HFpEF-like phenotypes with AF, tachycardia, or loss of effective ventricular filling	Targets a dynamic factor that worsens hemodynamics and neurohormonal activation

Abbreviations: AF—atrial fibrillation; CRT—cardiac resynchronization therapy; EF—ejection fraction; HFmrEF—HF with mildly reduced ejection fraction; HFrEF—HF with reduced ejection fraction; ICD—implantable cardioverter–defibrillator; LV—left ventricular; LVEF—left ventricular ejection fraction; QRS—QRS complex on electrocardiography.

## Data Availability

No new data were created or analyzed in this study. Data sharing is not applicable to this article.

## Abbreviations

The following abbreviations are used in this manuscript:

ACE	Angiotensin-converting enzyme
ACEi	Angiotensin-converting enzyme inhibitor(s)
AF	Atrial fibrillation
Ang II	Angiotensin II
AP-1	Activator protein 1
ARB	Angiotensin receptor blocker
ARNI	Angiotensin receptor–neprilysin inhibitor
AT1R	Angiotensin II type 1 receptor
ATP	Adenosine triphosphate
β1-AR	β1-adrenergic receptor
BNP	B-type natriuretic peptide
BP	Blood pressure
cAMP	Cyclic adenosine monophosphate
Ca^2+^	Calcium ion
cGMP	Cyclic guanosine monophosphate
CKD	Chronic kidney disease
CMR	Cardiac magnetic resonance
CTGF	Connective tissue growth factor
DAG	Diacylglycerol
DAMPs	Damage-associated molecular patterns
DNA	Deoxyribonucleic acid
E/e′	Ratio of early transmitral flow velocity to early diastolic mitral annular velocity
ECM	Extracellular matrix
EF	Ejection fraction
ERK	Extracellular signal-regulated kinase
GDMT	Guideline-directed medical therapy
GLS	Global longitudinal strain
GRK2	G protein-coupled receptor kinase 2
HF	Heart failure
HFimpEF	Heart failure with improved ejection fraction
HFmrEF	Heart failure with mildly reduced ejection fraction
HFpEF	Heart failure with preserved ejection fraction
HFrEF	Heart failure with reduced ejection fraction
HTN	Hypertension
IκBα	Inhibitor of nuclear factor kappa B alpha
IL	Interleukin
IP3	Inositol trisphosphate
JNK	c-Jun N-terminal kinase
LA	Left atrial
LTCC	L-type calcium channel
LV	Left ventricular
LVEF	Left ventricular ejection fraction
MAPK	Mitogen-activated protein kinase
MCU	Mitochondrial calcium uniporter
MMP	Matrix metalloproteinase
MRA	Mineralocorticoid receptor antagonist
mTOR	Mechanistic target of rapamycin
mPTP	Mitochondrial permeability transition pore
NADPH	Nicotinamide adenine dinucleotide phosphate (reduced form)
NCLX	Mitochondrial sodium/calcium exchanger
NE	Norepinephrine
NFAT	Nuclear factor of activated T cells
NF-κB	Nuclear factor kappa B
NLRP3	NOD-like receptor family pyrin domain containing 3
NO	Nitric oxide
NT-proBNP	N-terminal pro-B-type natriuretic peptide
PGC-1α	Peroxisome proliferator-activated receptor gamma coactivator 1-alpha
PI3K/Akt	Phosphoinositide 3-kinase/protein kinase B
PKA	Protein kinase A
PKC	Protein kinase C
PKG	Protein kinase G
PLC	Phospholipase C
RAAS	Renin–angiotensin–aldosterone system
ROS	Reactive oxygen species
RyR2	Ryanodine receptor 2
SERCA2a	Sarcoplasmic/endoplasmic reticulum Ca^2+^-ATPase 2a
sGC	Soluble guanylate cyclase
SGLT2	Sodium–glucose cotransporter 2
SGLT2i	Sodium–glucose cotransporter 2 inhibitor
SMAD	Mothers against decapentaplegic homolog signaling proteins
SNS	Sympathetic nervous system
SR	Sarcoplasmic reticulum
STAT3	Signal transducer and activator of transcription 3
TCA	Tricarboxylic acid cycle
TGF-β	Transforming growth factor beta
TIMP	Tissue inhibitor of metalloproteinase
TNF-α	Tumor necrosis factor alpha
VDAC	Voltage-dependent anion channel
VT	Ventricular tachycardia
